# Molecular Characterization of Melatonin Receptors in *Perccottus glenii* and Its Potential Roles in Melatonin-Mediated Antioxidant and Anti-Inflammatory Responses During Post-Freezing Recovery

**DOI:** 10.3390/antiox15080978

**Published:** 2026-08-06

**Authors:** Jiajun Zhou, Tianmei Liu, Zhaoyang Ning, Xiaoyu Zhao, Ye Huang, Kaitong Zhu, Xiangxin Kong, Weijie Mu

**Affiliations:** Laboratory of Fish Physiology and Resource Utilization, College of Life Science and Technology, Harbin Normal University, Harbin 150025, China; 16603097309@163.com (J.Z.); ltm159302@163.com (T.L.); 13275358746@163.com (Z.N.); 15845520679@163.com (X.Z.); fieldhwang@163.com (Y.H.); zkt757903@163.com (K.Z.); kongxiangxin0603@163.com (X.K.)

**Keywords:** melatonin receptor, *Perccottus glenii*, freezing and recovery process

## Abstract

In this study, we report for the first time the cloning and identification of three melatonin receptors (*PgMtnr1a*, *PgMtnr1b*, and *PgMtnr1c*) from the freeze-resistant fish *Perccottus glenii*, all of which encode typical GPCR proteins. These receptors are expressed widely in high-metabolism tissues such as the liver. During the freezing and recovery process at −2 °C, the expression of liver receptors exhibited dynamic changes: *PgMtnr1a* and *PgMtnr1c* were significantly upregulated during the middle of resuscitation, while *PgMTNR1A* reached its peak expression in the later stages, suggesting its involvement in stress repair. In vitro experiments confirmed that melatonin significantly enhances the activity of liver antioxidant enzymes in a receptor-dependent manner, an effect that can be inhibited by the antagonist luzindole. Anti-inflammatory analyses indicate that melatonin primarily inhibits inflammation-related genes through *Mtnr1a* and *Mtnr1b*. Furthermore, the overexpression of *PgMTNR1A* can synergistically activate ERK in the presence of melatonin, inhibit the JNK/p38 MAPK pathway, and downregulate the expression of *NF-κB* and *COX2*. Pathway inhibition experiments further validated that the MAPK/NF-κB axis, including ERK, JNK, and p38 pathways, mediates the anti-inflammatory effects of melatonin. This study systematically elucidates the molecular mechanisms by which the melatonin receptors of *P. glenii* play crucial antioxidant and anti-inflammatory roles during the later stages of freeze–thaw resuscitation, particularly by regulating the MAPK/NF-κB signaling axis through MTNR1A, thereby providing a novel basis for understanding the adaptation of vertebrates to extreme environments.

## 1. Introduction

Melatonin (*N*-acetyl-5-methoxytryptamine, Mel) is an evolutionarily conserved indoleamine neurohormone, well established for its role in regulating circadian rhythms across various biological groups [1,2,3,4,5,6]. Numerous studies have implicated it in processes such as antioxidant defense and apoptosis inhibition; however, the underlying mechanisms remain contentious. A significant body of research challenges the traditional view of melatonin acting as a direct free radical scavenger in vivo [1,7,8,9], with recent evidence also disputing its role in enhancing immuno-inflammatory responses [10]. Nevertheless, a prevailing perspective supports its function as an indirect antioxidant, although the precise molecular mechanisms, particularly its nuclear receptor-mediated inductive capabilities, remain unclear. Melatonin exerts its effects through both receptor-dependent and non-receptor-dependent pathways [3]. While its role as an indirect antioxidant is acknowledged, the exact molecular mechanisms, especially those mediated by its membrane receptors, continue to be elusive. In mammals, melatonin is predominantly synthesized by the pineal gland [11], yet the molecular basis of this synthesis remains unclear [1,12]. Increasing evidence suggests that melatonin exerts a cryoprotective effect, as demonstrated in cryopreservation studies involving sperm and tissues from goats (*Capra hircus*), crab-eating macaques (*Macaca fascicularis*), and chickens (*Gallus gallus*) [13,14,15]. Notably, these protective effects of melatonin typically require relatively high concentrations for administration, generally ranging from 100 µM to 1 mM [2,16]. However, its function in aquatic organisms remains poorly understood. Preliminary studies indicate that melatonin can enhance the antioxidant and immune responses of crustaceans and bony fish, such as the bronze featherback (*Notopterus notopterus*), thereby improving their stress resistance and survival rates [17]. Furthermore, the incorporation of melatonin into the feed has been demonstrated to increase the levels of antioxidant enzymes in the serum of eels (*Monopterus albus*) [18]. To gain a deeper understanding of the role of melatonin in fish, it is crucial to elucidate the distribution and regulatory mechanisms of its receptors [19].

Exploring the literature on the cloning of melatonin receptors across various species and the characterization methods employed during this process holds significant importance [20]. A comprehensive overview contrasting cloned sequences with in silico-predicted sequences, along with their gene structures, GC-rich regions, exon–intron boundaries, and cross-species conservation, has recently been established by Gautier et al. (2022) [21]. They systematically summarized available melatonin receptor sequences, highlighted annotation uncertainties, and emphasized the necessity of experimental cloning to validate computational predictions [21]. Melatonin signaling is mediated by G protein-coupled receptors (GPCRs), which are classified into three subtypes: MTNR1A (MT1), MTNR1B (MT2), and MTNR1C (Mel1c) [22]. While MT1 and MT2 are conserved across vertebrates, Mel1c is restricted to non-mammalian species, including fish, amphibians, and birds [23,24,25,26]. Three distinct melatonin receptor subtypes, namely MT1, MT2, and Mel1c, have been identified across various fish orders [19,27,28]. Melatonin exerts antioxidant and anti-inflammatory effects through both receptor-mediated and non-receptor-mediated pathways. Nonetheless, prior studies have predominantly concentrated on elucidating the functional role of melatonin, while the receptor-specific contributions to these mechanisms remain largely uncharacterized [29]. Research on melatonin receptors in teleosts has primarily focused on their roles in reproductive modulation and circadian behaviors [27,30], thereby leaving their potential involvement in stress adaptation, such as extreme cold tolerance, largely unexplored. Investigations into melatonin receptors in bovine granulosa cells have shown that silencing *MTNR1A* results in the downregulation of GPX4 and SOD1 expression, which impairs the granulosa cells’ response to exogenous melatonin. This indicates that MTNR1A mediates the positive effects of melatonin on the expression of antioxidant genes under physiological conditions [31]. However, numerous in vivo studies have employed pharmacological or elevated doses of melatonin that are known to internalize and inactivate melatonin receptors. For instance, Dong et al. (2026) reported that a high dose (20 mg/kg) of melatonin protects the liver from oxidative stress damage [32]. However, at such elevated dosages, the MT1 and MT2 receptors become internalized and functionally inactive, suggesting that this protective effect cannot be attributed to receptor-mediated signaling [32]. Therefore, the conclusion that this effect is mediated via MTNR1A necessitates cautious interpretation. Similarly, Li et al. (2020) demonstrated that melatonin activates the MAPK-ERK signaling pathway and inhibits inflammatory factors; however, the melatonin concentration utilized in that study may also be supraphysiological [33]. Without rigorous receptor blockade or knockdown experiments conducted at physiological doses, the receptor dependence of these anti-inflammatory effects remains to be validated further. While melatonin receptors are undoubtedly involved in certain anti-inflammatory and antioxidant processes at physiological levels, numerous studies employing high doses do not provide direct evidence for receptor-mediated mechanisms [33]. This indicates that enhancing anti-inflammatory capacity through melatonin receptor-mediated signaling is a key protective mechanism for the post-freezing recovery of *P. glenii*, a freeze-tolerant fish, rather than a general trait of cold resistance in fish. However, significant gaps persist in elucidating the receptor-dependent mechanisms and their temporal dynamics, particularly concerning the chronobiological regulation of melatonin receptor activity in fish. Furthermore, no studies have examined the role of melatonin receptors in freeze recovery mechanisms.

*Perccottus glenii*, a freeze-tolerant fish endemic to East Asia, exhibits remarkable resilience, surviving prolonged freezing at −2 °C with a 100% viability rate [34]. Laboratory experiments confirm that *P. glenii* maintains this survival rate after being frozen in ice at −2 °C for 24 h [35]. Pioneering multi-omics research by Jiang et al. (2023) established the chromosomal-level genome of *P. glenii* and revealed that its freeze tolerance relies on coordinated, tissue-specific regulation of genes and metabolites involved in hypometabolism, cellular stress responses (including antioxidant defense), and cryoprotectant accumulation [36]. Our previous research has established a foundation for understanding this phenomenon by documenting the successful recovery of *P. glenii* from freezing [37], observing elevated melatonin levels during this recovery process [38], and identifying the activation of critical immune and metabolic pathways [37]. Most recently, we demonstrated that melatonin plays a time-dependent role in post-freeze recovery by modulating autophagy, mitochondrial function, and antioxidant processes in the liver, with its effects involving receptor-mediated activation of the PKC/ERK pathway [38]. To date, the specific molecular mechanisms underlying melatonin’s capacity as an antioxidant, particularly through its membrane receptors, remain incompletely elucidated in *P. glenii*. The liver, a central organ in metabolic and antioxidant regulation, serves as a key site for this investigation [39]. Currently, the dynamics of melatonin receptor mRNA expression during the recovery from freezing in *P. glenii*, along with the subtype-specific functions and downstream time-dependent signaling pathways involved, remain unclear. Despite the progress made, critical gaps persist: (1) the expression profile of melatonin receptor subtypes in *P. glenii* tissues is unknown; (2) the receptor-dependent molecular mechanisms underlying melatonin-induced antioxidant and anti-inflammatory gene expression remain poorly understood. Most previous conclusions rely on indirect evidence, and studies utilizing supraphysiological melatonin concentrations cannot reliably attribute effects to membrane receptor signaling, as receptors are inactive at such doses; and (3) the temporal dynamics of receptor-mediated signaling during freeze recovery have yet to be investigated.

This study investigates the role of melatonin (MLT) in the recovery of *P. glenii* from freezing and elucidates the underlying mechanisms. The primary objectives are (1) to characterize the expression of *PgMtnr1a*, *PgMtnr1b*, and *PgMtnr1c* in the tissues of *P. glenii*; (2) to analyze their expression in the liver using quantitative PCR (qPCR) and Western blotting; (3) to determine whether melatonin induces the expression of antioxidant enzymes; (4) to assess the impact of various concentrations of melatonin on the expression of anti-inflammatory and antioxidant genes; (5) to utilize melatonin receptor antagonists to evaluate the effects of melatonin; and (6) to elucidate the downstream signaling pathways, particularly the MAPK/NF-κB axis, via *PgMtnr1a* overexpression and pathway inhibition.

## 2. Material and Methods

### 2.1. Animals, Acclimation Conditions, and Sampling Schedule

Adult *P. glenii* (mean body weight: 21.32 ± 2.45 g) were collected from the Songhua River in Heilongjiang Province, China, and transported to the laboratory under oxygenated conditions. The fish were acclimated for 14 days in recirculating aquaculture systems with a capacity of 200 L, maintained at 16.0 ± 1.5 °C and equipped with continuous biofiltration (pH 7.5; dissolved oxygen 7.0 mg/L). The 12 L: 12D photoperiod is commonly used as a baseline for natural light in animal experiments [40,41]. This photoperiod was kept constant across all groups to control for its confounding effects on melatonin signaling. During the acclimation period, the fish were fed live earthworms ad libitum and were fasted for 24 h prior to experimentation. To minimize the influence of circadian rhythms on melatonin receptor expression, all experimental manipulations and tissue samplings were strictly performed during the early light phase (08:00–10:00), ensuring that all groups were compared at the identical circadian phase. All procedures were approved by the Animal Ethics Committee of Harbin Normal University (Protocol HNUARIA2023007).

### 2.2. Recovery from Freezing and Sampling

A total of 180 fish were randomly allocated into control and experimental groups, with 30 fish per tank and three replicates per group. The control group was maintained at a constant temperature of 16.0 ± 1.5 °C throughout the experiment. In contrast, the experimental group underwent a gradual temperature reduction from 16 °C to −2 °C at a rate of 1 °C per hour over 24 h, followed by rewarming to 4 °C. The initiation of recovery (0 h) was defined as the moment when the water temperature reached 4 °C. Fish from the experimental group were sampled at 0, 4, 8, and 12 h post rewarming, with 12 fish at each time point. Following anesthesia with MS-222, heart, liver, and spleen tissues were dissected, flash-frozen in liquid nitrogen, and stored at −80 °C. For baseline tissue distribution profiling, organs were collected from untreated healthy fish (*n* = 6).

### 2.3. Gene Cloning

Total RNA was extracted from the tissues using RNAiso Plus (Takara, Dalian, China). The quality and quantity of the extracted RNA were assessed using a spectrophotometer. For routine cDNA synthesis, the RNA was processed with the PrimeScript™ FAST RT reagent Kit, which includes a gDNA Eraser (Takara, Dalian, China). The sequences of *mtnr1a*, *mtnr1b*, and *mtnr1c* were retrieved from the *P. glenii* genome (NCBI GCA_024416835.2) using BLAST (version BLAST+ 2.16.0, accessed on 2 April 2025) against zebrafish orthologs (E-value < 1 × 10^−10^). However, since the genome-derived sequences lacked the complete full open reading frames (ORFs), rapid amplification of cDNA ends (RACE) was performed to obtain the full-length transcripts. For RACE, first-strand cDNA was synthesized using the SMARTer^®^ RACE 5′/3′ Kit (TaKaRa, Kusatsu, Shiga, Japan) according to the manufacturer’s instructions. Gene-specific RACE primers were designed based on the partial genomic sequences (Table 1), and the RACE PCR products were amplified under the following conditions: 94 °C for 30 s, 68 °C for 30 s, and 72 °C for 3 min for 30 cycles. The resulting RACE products were gel-purified, cloned into the pMD18-T vector (TaKaRa, Japan), and sequenced. Based on the assembled full-length sequences, ORF-specific primers were then designed to amplify the complete coding sequences (CDSs) of *mtnr1a*, *mtnr1b*, and *mtnr1c* (Table 1). The full-ORF PCR was performed using PrimeSTAR^®^ HS DNA Polymerase (TaKaRa, Japan) under the following thermocycling conditions: 95 °C for 5 min, followed by 34 cycles of 95 °C for 30 s, 60 °C for 30 s, and 72 °C for 2 min, concluding with 72 °C for 8 min. The purified full-ORF PCR products were cloned into the pMD18-T vector and transformed into TOP10 E. coli. Selected positive clones were sequenced by Ruibiotech Co., Ltd. (Qingdao, China). The complete transcript sequences of *mtnr1a*, *mtnr1b*, and *mtnr1c* have been deposited in GenBank under the accession numbers PV433192, PV424064, and PV433193, respectively.

### 2.4. Bioinformatics Analysis

The protein functional domains were predicted using SMART (http://smart.embl.de/, accessed on 31 January 2024). The web-based ExPASy tool (https://www.expasy.org/, accessed on 31 January 2024) was utilized to assess the calculated molecular weight and theoretical isoelectric point. For phylogenetic analysis, the predicted amino acid sequences were aligned using CLUSTAL W (version 2.1) and CLUSTAL X, and the resulting alignments were curated using GBLOCKS with less stringent parameters. A phylogenetic tree was constructed using the neighbor-joining (NJ) method in MEGA 10 software, based on pairwise Poisson correction distances, with the reliability of the branching order assessed by 1000 bootstrap replicates. The resulting tree was rooted using sequences from *Callorhinchus milii*, *Megalops cyprinoides*, and *Thalassophryne amazonica* as the outgroups. The accession numbers of all reference sequences used in the phylogenetic analysis are listed in Supplementary Table S1.

### 2.5. In Vivo Analysis

#### 2.5.1. Real-Time Quantitative PCR (RT-qPCR)

Liver tissue samples were collected at recovery time points of 0, 4, 8, and 12 h post freezing, with six fish sampled at each time point. All samples were immediately frozen in liquid nitrogen for subsequent gene and protein quantification. Total RNA was extracted using the RNAiso Plus reagent (Takara, Dalian, China). The quantity and purity of RNA were assessed using A260/280 ultraviolet spectrophotometry, while its integrity was evaluated through 1.0% agarose gel electrophoresis. The RNA integrity was confirmed by the clear presence of intact 28S and 18S rRNA bands without smearing; the A260/280 and A260/230 ratios for all samples ranged from 1.85 to 2.05 and 2.10 to 2.30, respectively. One microgram of RNA was transcribed to cDNA using the RT reagent Kit with gDNA Eraser (Takara Bio, Dalian, China). The resulting cDNA was diluted 10-fold with nuclease-free water before qPCR. Quantitative real-time PCR (qPCR) was performed using the CFX96 system (Bio-Rad, Hercules, CA, USA) with TB Green^®^ Premix Ex Taq™ II (Takara Bio, China). The qPCR reaction volume was 20 μL, containing 0.4 μM of each gene-specific primer. The specificity of the PCR products was confirmed by melting curve analysis. The cycling protocol consisted of an initial denaturation step at 95 °C for 30 s, followed by 40 cycles of 95 °C for 5 s and 60 °C for 30 s. A melting curve analysis was conducted to verify amplification specificity. Primer efficiencies (90–110%) were validated by standard curves. Relative expression was calculated via the 2^−ΔΔCt^ method [42] with β-actin as the reference, using the liver sample (for tissue profiling), the R0 group (for treatment assays) or the OE-MTNR1A (for the primary cell assays) as the calibrator.

#### 2.5.2. Western Blot

Proteins were extracted using RIPA lysis buffer (Beyotime, Shanghai, China), supplemented with 1 mM PMSF protease inhibitor (Beyotime, China). The procedure involved the following steps: 20 mg of tissue was taken, and 200 μL of lysis buffer was added. The mixture was homogenized on ice using a homogenizer for 3 min. The homogenate was then incubated on ice for 30 min, with vortexing every 10 min during the incubation period. Subsequently, the sample was centrifuged at 12,000 rpm for 10 min at 4 °C. The supernatant was collected, and the protein concentration was determined using the BCA protein quantification kit (Beyotime, China) following the manufacturer’s instructions. The protein sample was mixed with 5 × SDS loading buffer (Beyotime, China) at a ratio of 1:4, boiled at 100 °C for 7 min, and stored at −40 °C.

For Western blot analysis, a 12% SDS-PAGE gel was prepared using the SDS-PAGE gel rapid preparation kit (Beyotime, China). The protein sample (20 μg per well) and a protein marker (10–180 kDa, Beyotime, China) were loaded onto the gel, and electrophoresis was conducted at 20 V for 10 min, 80 V for 30 min, and 120 V for 40–50 min. The proteins were transferred to a PVDF membrane (Beyotime, China) using a wet transfer system at a current of 120 mA for 40 min. The membrane was blocked with QuickBlock™ Western blocking solution (Beyotime, China) for 20 min, and then incubated overnight at 4 °C with primary antibodies (β-actin, 1:5000, RRID: AB_2768234; MTNR1A, 1:3000, RRID: AB_2759878; ABclonal, Wuhan, China; NF-κB, 1:1500, RRID: AB_3752910; COX2, 1:3000, RRID: AB_3752911; Beyotime, China) diluted in antibody diluent (Beyotime, China). Following washing with TBST, the membrane was incubated with HRP-labeled secondary antibodies (1:50,000; Beyotime, China) for 60 min. After another washing step with TBST, the protein bands were visualized using a chemiluminescence kit (Beyotime, China) and imaged with a gel imaging system.

### 2.6. In Vitro Analysis

#### 2.6.1. Effects of Melatonin Combined with Receptor Antagonists on Antioxidant Enzymes in Liver Tissue In Vitro

On the day of the experiment, *P. glenii* (*n* = 6) were anesthetized and euthanized after a 24-h fasting period. Liver fragments (approximately 10 mg each) were aseptically excised and cultured in 24-well plates containing Hanks medium (1 mL/well) under conditions of 5% CO_2_ at 20 °C. The fragments from each liver were randomly distributed into different treatment groups.

The groups were as follows: (1) Control (medium only); (2) Luzindole alone (10^−7^ M or 10^−6^ M); (3) 4-P-PDOT alone (10^−7^ M or 10^−6^ M); (4) Prazosin alone (10^−7^ M or 10^−6^ M); (5) Melatonin alone (10^−9^ M or 10^−8^ M); (6) Melatonin + Luzindole; (7) Melatonin + 4-P-PDOT; (8) Melatonin + Prazosin. The concentrations of melatonin (10^−9^ M, 10^−8^ M) and receptor antagonists (10^−7^ M, 10^−6^ M) were based on preliminary experiments, where these parameters were confirmed to induce detectable changes in antioxidant enzyme activity. Luzindole (L420793), 4-P-PDOT (P1493171) and Prazosin (P305290) were procured from Shanghai Aladdin Biochemical Technology Co., Ltd., Shanghai, China.

At the end of culture period, liver samples were collected, quickly frozen in liquid nitrogen, and stored at −80 °C until analysis. The activities of superoxide dismutase (SOD), catalase (CAT), and glutathione peroxidase (GPx) in the homogenates of liver tissues were measured using commercial kits (Nanjing Jiancheng Co., Ltd., Nanjing, China). The enzyme activities were normalized to the protein content of the samples, as determined by the Bradford method, and expressed as units per milligram of protein (U/mg prot).

#### 2.6.2. Primary Hepatocyte Culture and Treatment

Cultured primary hepatocytes were collected using our previously reported method [38]. In brief, primary hepatocytes were isolated via collagenase IV (1 mg/mL) digestion (2200 rpm/2 min centrifugation). After 40 μm filtration, cells were seeded in DMEM (10% FBS + antibiotics) at a density of 4 × 10^5^ cells/well (24-well plates) and maintained at 20 °C. Cells were randomly assigned to the following groups (each group contained six biological replicates):

1. Blank Control Group: Untransfected cells cultured in medium alone (Control).
2. Melatonin Treatment Groups: Mel (concentration of 10^−9^ M Melatonin).
3. Luzindole Alone Treatment Groups: (concentration of 10^−7^ M Luzindole).
4. 4P-P-DOT Alone Treatment Groups: (concentration of 10^−7^ M 4P-P-DOT).
5. Prazosin Alone Treatment Groups: (concentration of 10^−7^ M Prazosin).
6. Melatonin with Luzindole Treatment Groups: Mel + Luzindole (concentration of 10^−9^ M Melatonin with 10^−7^ M Luzindole).
7. Melatonin with 4P-P-DOT Treatment Groups: Mel + 4P-P-DOT (concentration of 10^−9^ M Melatonin with 10^−7^ M 4P-P-DOT).
8. Melatonin with Prazosin Treatment Groups: Mel + Prazosin (concentration of 10^−9^ M Melatonin with 10^−7^ M Prazosin).
9. Melatonin Alone Treatment Groups: Mel (concentration of 10^−8^ M).
10. Luzindole Alone Treatment Groups: (concentration of 10^−6^ M Luzindole).
11. 4P-P-DOT Alone Treatment Groups: (concentration of 10^−6^ M 4P-P-DOT).
12. Prazosin Treatment Groups: (concentration of 10^−6^ M Prazosin).
13. Melatonin with Luzindole Treatment Groups: Mel + Luzindole (concentration of 10^−8^ M Melatonin with 10^−6^ M Luzindole).
14. Melatonin with 4P-P-DOT Treatment Groups: Mel + 4P-P-DOT (concentration of 10^−8^ M Melatonin with 10^−6^ M 4P-P-DOT).
15. Melatonin with Prazosin Treatment Groups: Mel + Prazosin (concentration of 10^−8^ M Melatonin with 10^−6^ M Prazosin).

Cells were exposed to melatonin with receptor antagonists for 24 h. For qRT-PCR analysis, specific primers (Table 1) were designed for target genes (*PgMtnr1a*, *PgMtnr1b*, *PgMtnr1c*, *Nf-κb* and *Cox2*). β-actin was used as a reference gene. The control group was served as the reference control for fold-change determination using the 2^−ΔΔCt^ method.

#### 2.6.3. PgMtnr1a Overexpression and Cell Experiments

HEK293T cells were cultured in DMEM supplemented with 10% fetal bovine serum, 100 U/mL penicillin, and 100 μg/mL streptomycin at 37 °C in a humidified atmosphere containing 5% CO_2_. Upon reaching 70–80% confluence, the cells were transfected using Lipofectamine 3000 reagent. The use of HEK293T cells in this study follows the widely accepted “dual-platform validation paradigm” in aquatic biology research, where mammalian cell lines serve as an efficient biochemical platform for mechanistic screening and target-species primary cells are used for functional confirmation [43,44,45]. Specifically, HEK293T cells were employed here to rapidly and efficiently address the mechanistic question of whether MTNR1A possesses the intrinsic capacity to activate MAPK/NF-κB signaling at the molecular level, taking advantage of their high transfection efficiency and clean genetic background. All key functional conclusions regarding MTNR1A-mediated receptor functions in *P. glenii* were independently validated in fish primary hepatocytes and liver tissue. To overexpress MTNR1A, the full-length open reading frame (ORF) along with its native 5′ and 3′ untranslated regions was cloned into the pcDNA3.1 (+) vector, resulting in the generation of the pcDNA3.1-MTNR1A plasmid. This construct was utilized for transient transfection in all subsequent experiments, while the empty pcDNA3.1 (+) vector served as the transfection control. All experiments were conducted with three independent biological replicates. To systematically analyze the effect of MTNR1A overexpression and melatonin treatment, the experiment was structured as follows:

Baseline Expression Controls: These groups were established to assess the basal and overexpression levels of MTNR1A without any melatonin treatment.

Blank Control Group: Untransfected cells cultured in standard medium.

Empty Vector Control Group: Cells transfected with the empty pcDNA3.1 (+) vector.

MTNR1A Overexpression Group: Cells transfected with the pcDNA3.1-MTNR1A plasmid.

Melatonin Treatment Groups: To evaluate the specific effect of melatonin (10^−5^ M, 24-h treatment), the three baseline groups above were treated in parallel.

Control + Melatonin (Control + Melatonin)

Empty Vector + Melatonin (Empty vector + Melatonin)

MTNR1A Overexpression + Melatonin (OE-MTNR1A + Melatonin)

In the present analysis, the primary comparison was made between the effects of melatonin on different genetic backgrounds, specifically between OE-MTNR1A + Melatonin and OE-MTNR1A. Additionally, comparisons with empty vector controls subjected to melatonin treatment were conducted to validate the receptor-dependent nature of the observed effects.

Pathway Inhibitor Groups: To investigate the signaling mechanisms, cells from the three genetic backgrounds (Control, Empty vector, OE-MTNR1A) were pretreated for 1 h with a specific pathway inhibitor (10^−5^ M of JNK inhibitor SP600125, p38 inhibitor SB202190, or ERK inhibitor PD98059) followed by co-incubation with 10^−5^ M melatonin for 24 h [40]. These groups are designated as, for example, OE-MTNR1A + Melatonin + Pathway inhibitor (OE-MTNR1A + Melatonin + Pathway inhibitor).

For qRT-PCR analysis, total RNA was extracted from all groups, and expression levels of target genes (*MTNR1A*, *ERK*, *p38*, *COX2*, *JNK* and *NF-κB*) were quantified using gene-specific primers (Table 1). Human-specific primers were also designed based on reference sequences (Table S1).

#### 2.6.4. Quantitative PCR Analysis

To detect the expression levels of key genes in tissues, including primary hepatocytes and HEK293 cells subjected to various treatments, we performed RNA extraction followed by quantitative real-time polymerase chain reaction (qRT-PCR). Total RNA was extracted from each group using the RNAiso Plus reagent, and complementary DNA (cDNA) was synthesized using the PrimeScript™ RT kit. The RT-qPCR was conducted with TB Green^®^ Premix (Takara Bio, China) on a CFX96 system (Bio-Rad, USA). The cycling conditions were as follows: 95 °C for 30 s, followed by 40 cycles of 95 °C for 5 s and 60 °C for 30 s. Primer efficiencies, which ranged from 90% to 110%, were validated using standard curves. Beta-actin was utilized as the reference gene, and data were analyzed using the 2^−ΔΔCt^ method.

#### 2.6.5. Western Blot Analysis

Primary hepatocytes from each treatment group in Section 2.6.2 were collected after culture, lysed with RIPA lysis buffer (containing protease inhibitors) on ice for 30 min, and then centrifuged at 12,000× *g* for 15 min at 4 °C. The supernatant was collected for further analysis. The protein concentration was determined using a bicinchoninic acid (BCA) protein assay kit (Beyotime, China), in accordance with the manufacturer’s instructions. A 12% (vol/vol) SDS-polyacrylamide gel was employed to separate 20 μg of total protein on a polyvinylidene difluoride membrane (Bio-Rad, USA). The membranes were blocked with 5% skim milk in Tris-buffered saline containing 0.1% Tween 20 (TBST) overnight at 4 °C. Following this, the membranes were washed with TBST for 10 min. Subsequently, the membranes were incubated for 2 h with primary antibodies specific to NF-κB (A27463, Beyotime, China) and COX2 (AF1924, Beyotime, China). After three washes with TBST buffer, each lasting 10 min, goat anti-mouse IgG HRP (1:4000) was applied as a secondary antibody and incubated at room temperature for 1 h. The membranes were then washed three times with TBST buffer for 10 min each. Finally, the epitope was visualized using a BeyoECL Plus kit (Beyotime, China), and quantification was performed using the ImageJ software package (Version 1.43, Bethesda, MD, USA).

### 2.7. Statistical Analysis

**Figure 1:**
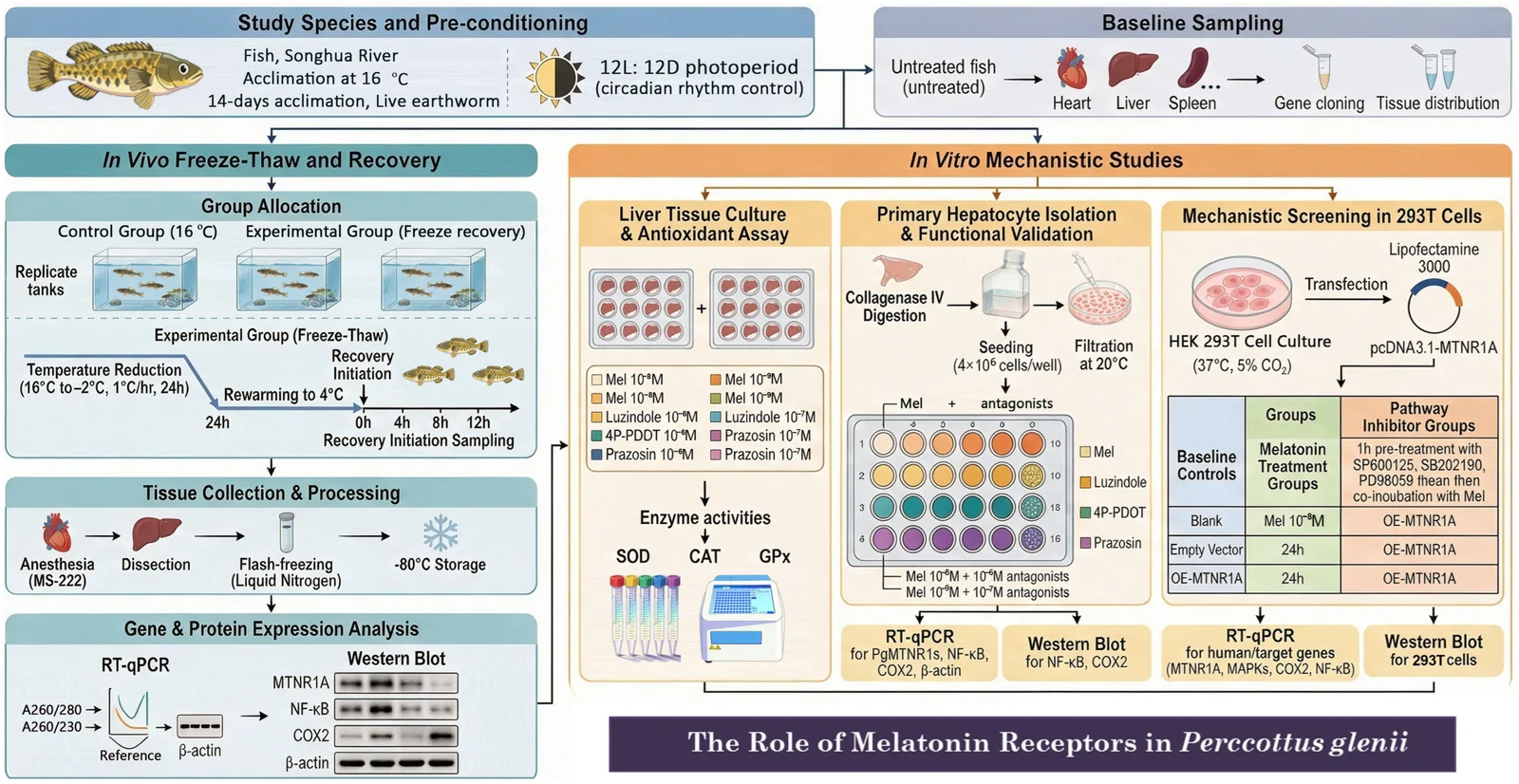
A graphic visualization of the workflow in our study.

Normality was assessed using the Shapiro–Wilk test, and homoscedasticity (equality of variances) was assessed using the Brown–Forsythe test. Data are presented as the mean ± SEM. For single-factor comparisons (tissue distribution, temporal expression during recovery, and single-treatment assays), one-way ANOVA with Tukey’s post hoc test was performed. For pharmacological experiments involving two categorical independent variables (treatment type and melatonin concentration), two-way ANOVA was used to evaluate the main effects of each factor and their interaction; when a significant interaction was detected, post hoc comparisons were conducted using Tukey’s test. Statistical analysis was conducted using GraphPad Prism (version 11.0) and SPSS software (version 23.0). Significance was considered at *p* < 0.05. The main experimental workflow of this study is shown in Figure 1.

## 3. Results

### 3.1. Full-Length cDNA Cloning and Comparative Analysis of PgMtnr1a, PgMtnr1b, and PgMtnr1c

**Figure 2:**
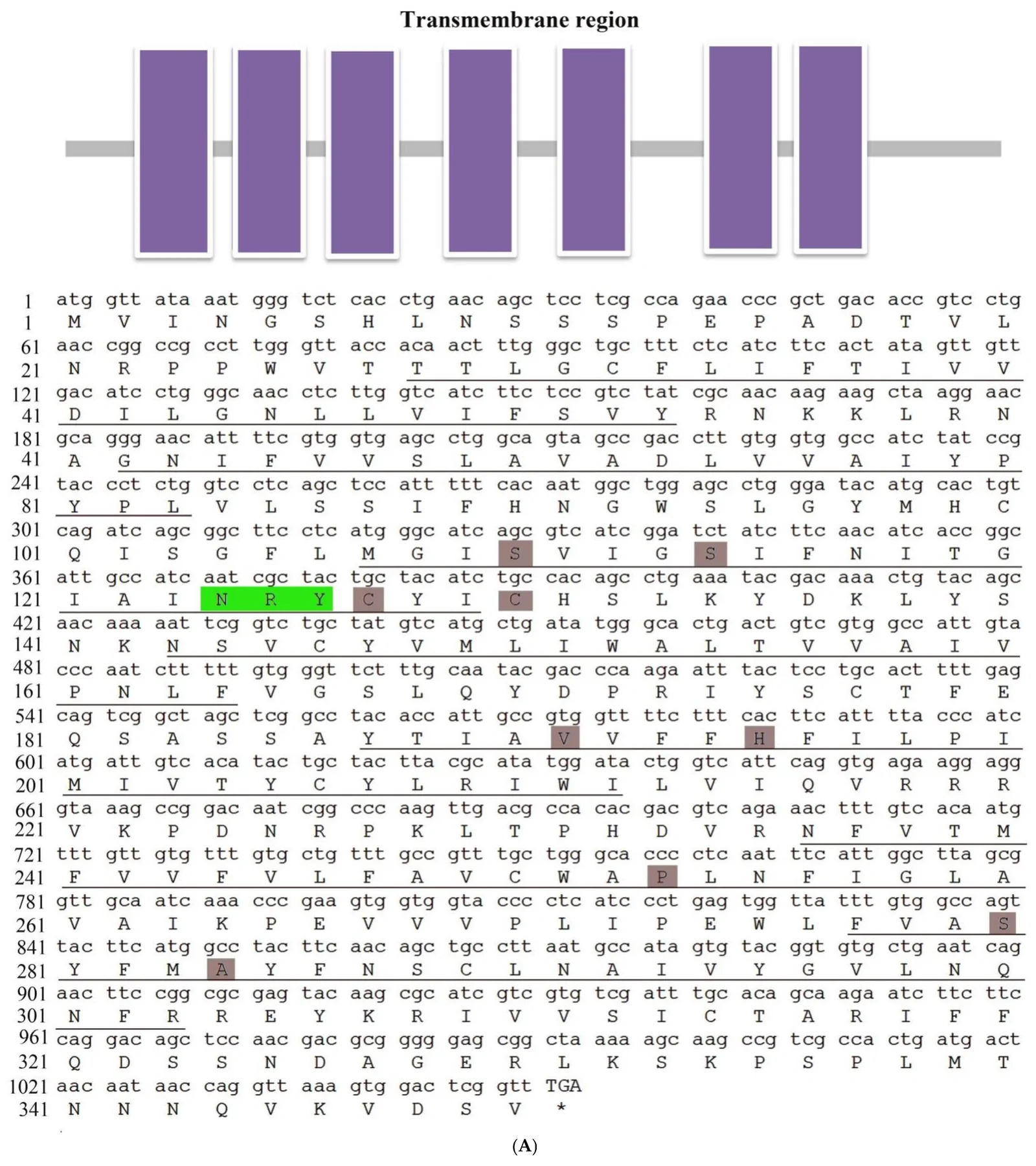

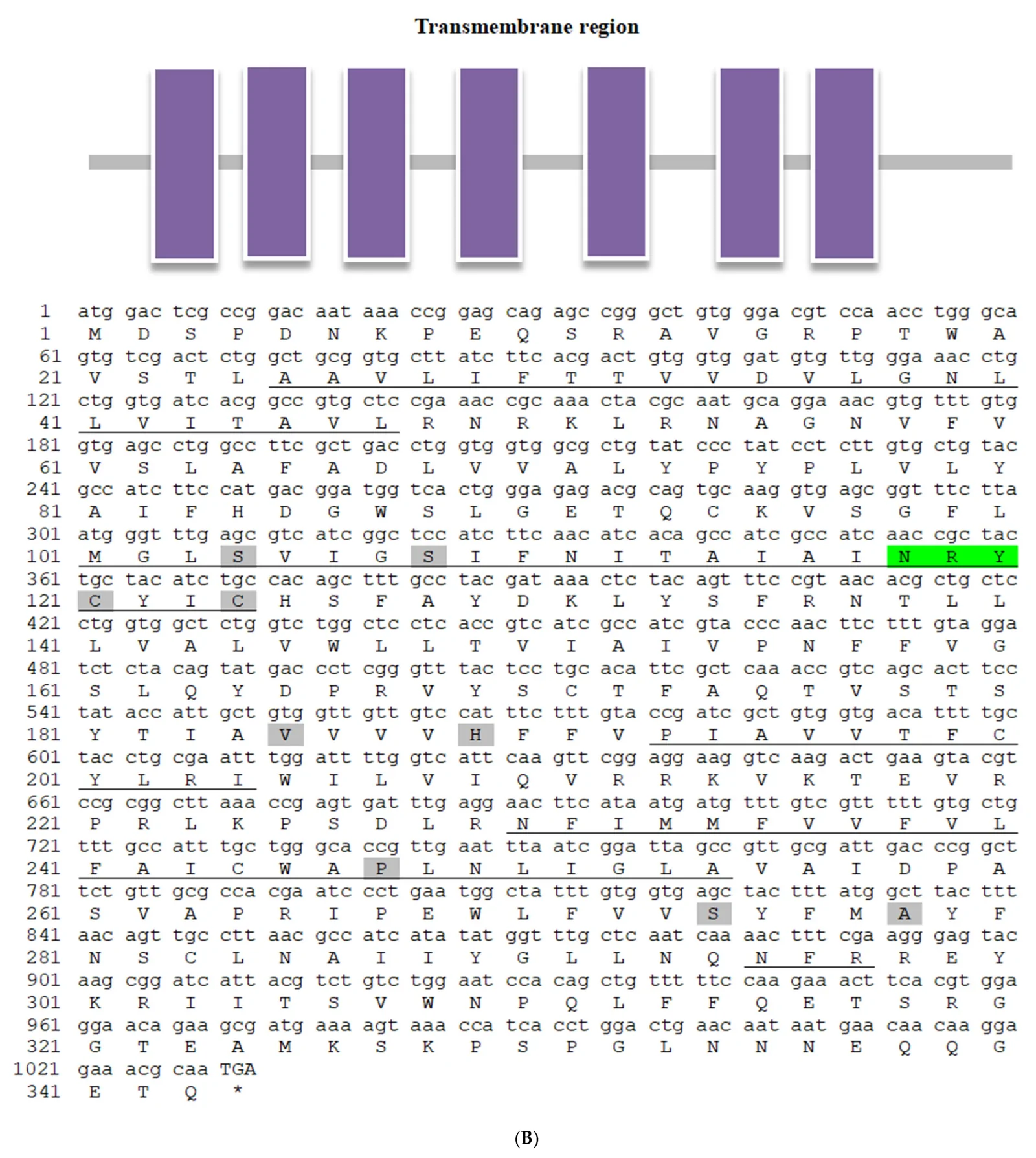

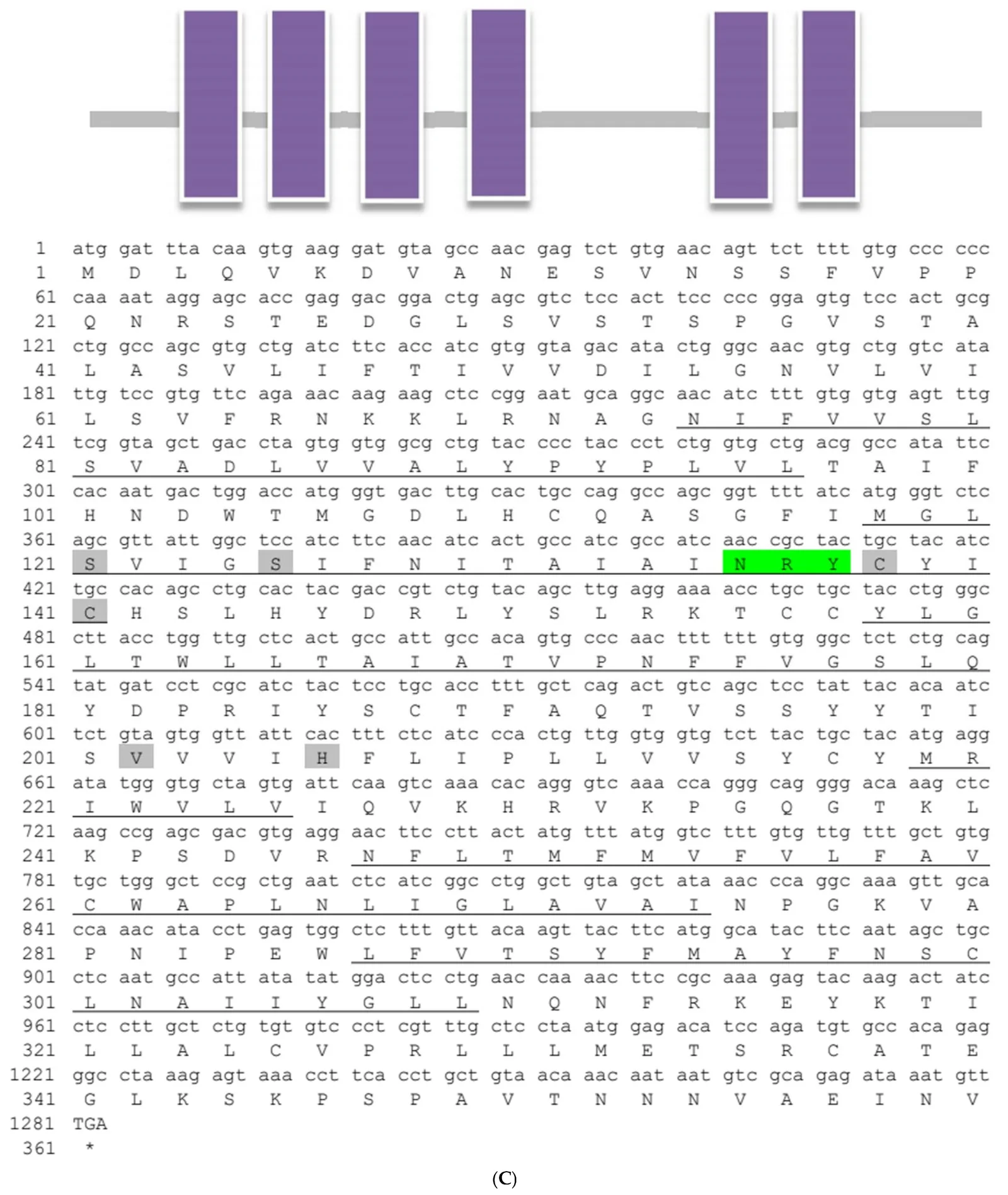
Deduced amino acid sequence of PgMtnr1A (**A**), PgMtnr1B (**B**) and PgMtnr1C (**C**). The sole sequence is the last listed. The transmembrane domains are underlined (sequentially from I to VII). Amino acids known to be important for the proper function of mammalian Mtnr1A are on a gray background. The green box shows the conserved NRY motif which is known to be involved in G protein coupling.

The complete deduced amino acid sequences of PgMTNR1A, PgMTNR1B, and PgMTNR1C are illustrated in Figure 2. The open reading frame (ORF) of PgMTNR1A encodes a protein comprising 350 amino acids (aas), with a predicted molecular mass of 39.62 kDa and a theoretical isoelectric point of 9.27. We confirmed that PgMtnr1A possesses seven transmembrane domains and an NRY motif (Figure 2A). This protein contains a critical functional domain analogous to those found in mammals, and its structural features classify it within the melatonin group of the prototypic G protein-coupled receptor family. According to the NCBI Align Sequences Protein BLAST, PgMTNR1A exhibits a sequence identity ranging from 93.50% to 97.43% with other vertebrate homologs, with the highest identity (97.43%) observed with MTNR1A from *Morone saxatilis* (Accession No. XP_035515238.1). The ORF of PgMTNR1B encodes a protein of 343 amino acids, with a predicted molecular mass of 38.69 kDa and a theoretical isoelectric point of 9.53. We verified that PgMtnr1B contains seven transmembrane domains and an NRY motif (as illustrated in Figure 2B). The PgMtnr1B protein harbors a significant functional domain analogous to that of mammalian species. PgMTNR1C encodes a protein of 264 amino acids (Figure 2C). Through NCBI Align Sequences Protein BLAST analysis, PgMTNR1B displays a sequence identity ranging from 80.91% to 92.42% in comparison with other vertebrate homologs, with the maximum identity (92.42%) detected between PgMTNR1B and that from *Periophthalmus magnuspinnatus* (Accession No. XP_033832853.2). All three receptor types exhibit seven transmembrane domains, which are characteristic features of G protein-coupled receptors (Figure 2).

**Figure 3:**
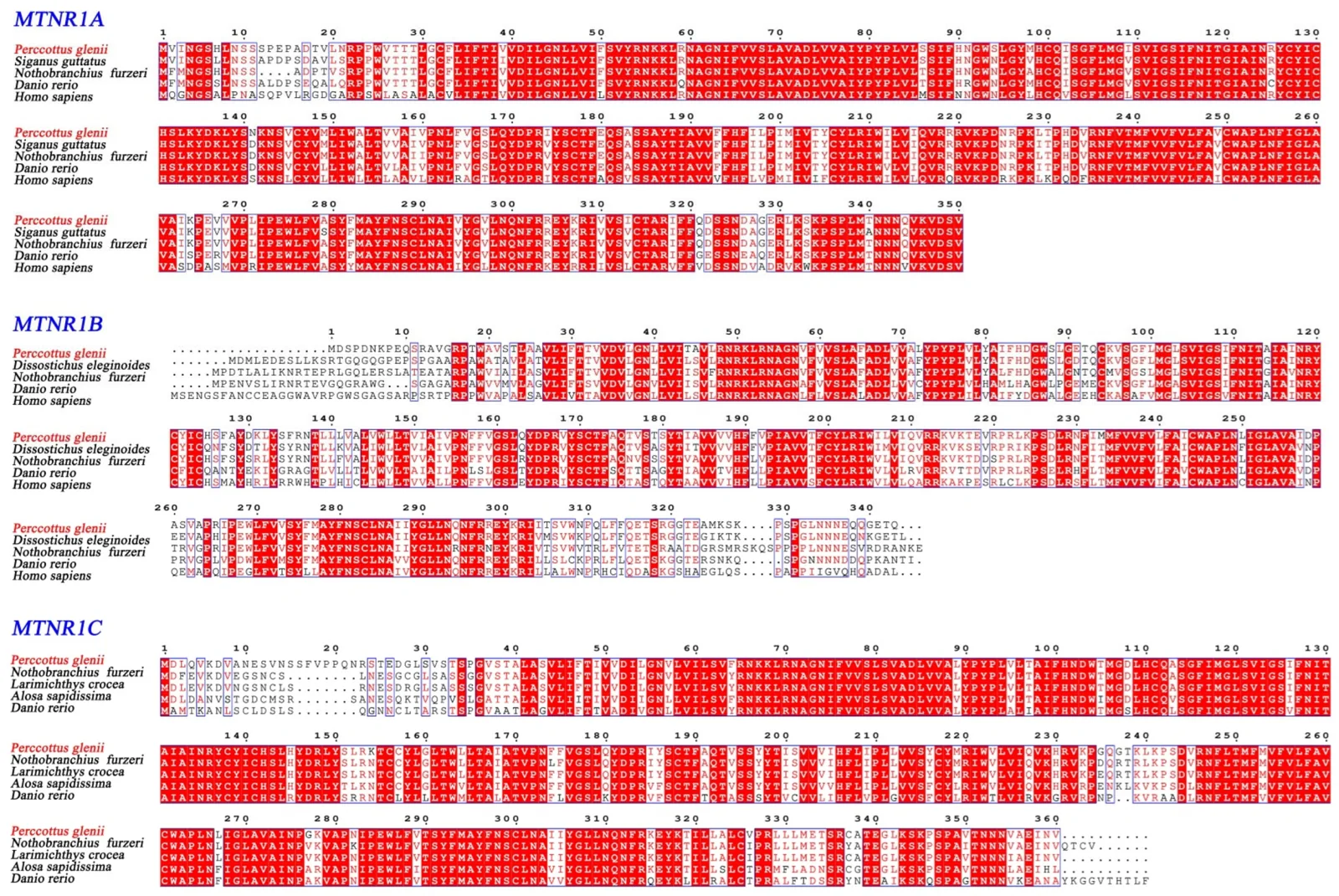
Alignment of the *P. glenii* melatonin receptors’ amino acid sequence with those of other teleosts and human. Gaps introduced to optimize the alignment are indicated by dots (.).

Within these receptors, specific motifs typical of melatonin receptors were identified, including an NRY motif located directly downstream of transmembrane domain III. Similarity analysis indicated that the deduced amino acid sequence of the melatonin receptors in *P. glenii* is highly conserved when compared to those in teleosts and humans (Figure 3). Homology analysis revealed that the derived amino acid sequence of the melatonin receptor in *P. glenii* exhibits significant conservation with melatonin receptors in other bony fish and reported human receptors, demonstrating a high degree of homology.

### 3.2. Phylogenetic Analysis

**Figure 4:**
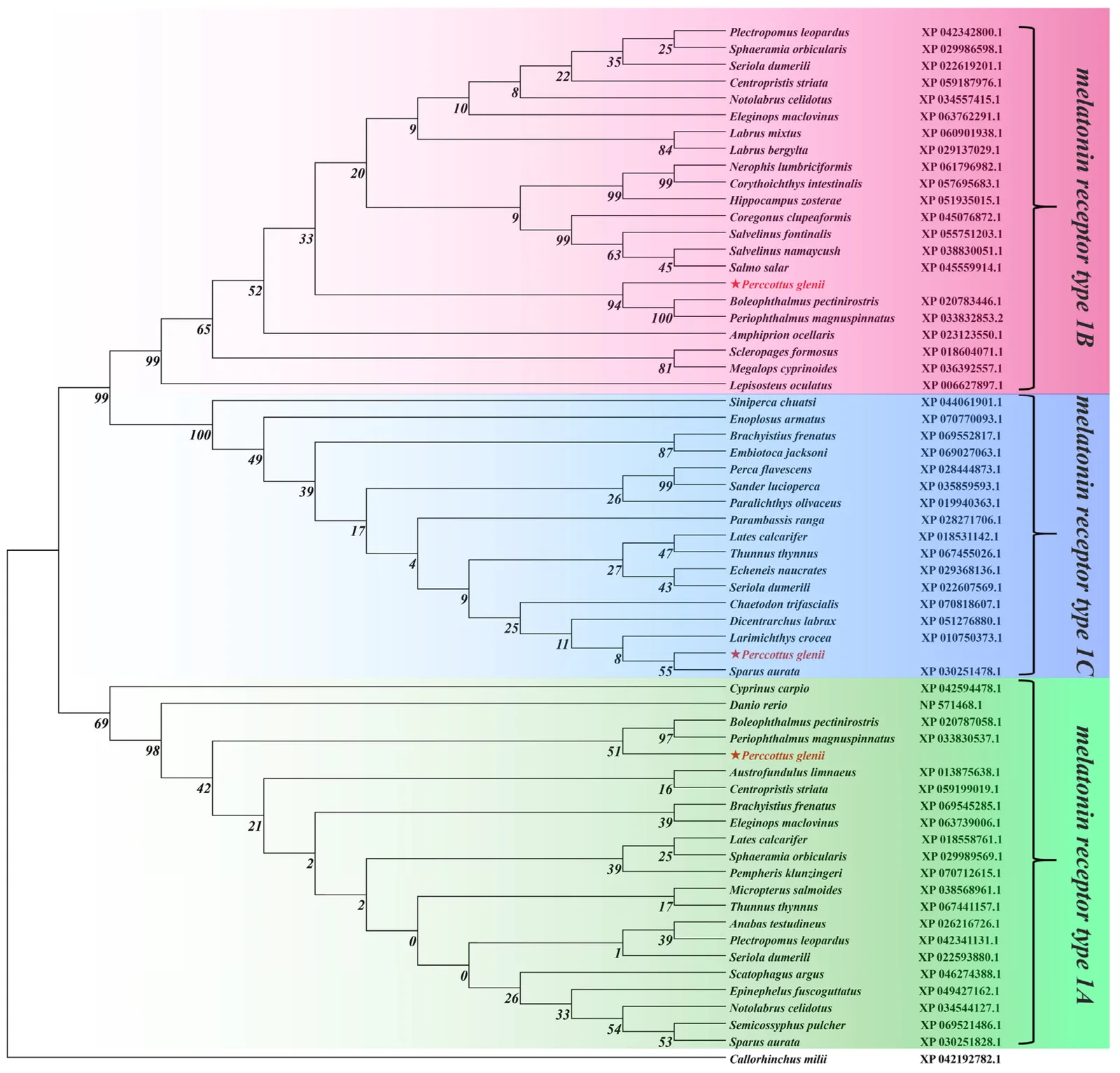
The phylogenetic relationships of MTNR1A, MTNR1B and MTNR1C. A neighbor-joining phylogenetic tree was constructed based on the analysis of sixty-two amino acid sequences of MTNR by MEGA10. The amino acid sequences used are shown in Table S2.

Alignment of the full length of amino acid sequences from different species is shown in Figure 4. This study elucidates the phylogenetic analysis and evolutionary relationships among the melatonin receptors PgMTNR1A, PgMTNR1B, and PgMTNR1C (Figure 4). A Neighbor-Joining (NJ) phylogenetic tree was constructed utilizing 62 fish species’ MTNR protein sequences. This dataset incorporated homologous sequences from representative vertebrate classes, including Actinopterygii, Amphibia, Reptilia, Aves, and Mammalia. The melatonin receptors are categorized into three distinct subtypes: MTNR1A, MTNR1B, and MTNR1C (Figure 4). Phylogenetic analysis indicated that MTNR genes predominantly clustered according to their respective vertebrate classes, aligning with established taxonomic classifications. The PgMTNR1A formed a well-supported clade with sequences from *Periophthalmus magnuspinnatus* and *Boleophthalmus pectinirostris*. This clade, comprising Perccottus, Periophthalmus, and Boleophthalmus, was sister to a clade containing salmonids (*Salmo salar*, *Oncorhynchus tshawytscha*). The PgMTNR1B sequence demonstrated the highest similarity to that of *Sparus aurata* and clustered within a larger clade of marine teleosts, including *Dicentrarchus labrax* and *Larimichthys crocea*, indicating a common ancestor. Finally, this study suggests that the MTNR1C subtype is present in both fish and amphibians, with the PgMTNR1C subtype positioned in a clade adjacent to sequences from species such as *Plectropomus leopardus* and *Anabas testudineus*.

### 3.3. Tissue Distribution of PgMtnr1a, PgMtnr1b, and PgMtnr1c mRNA

**Figure 5:**
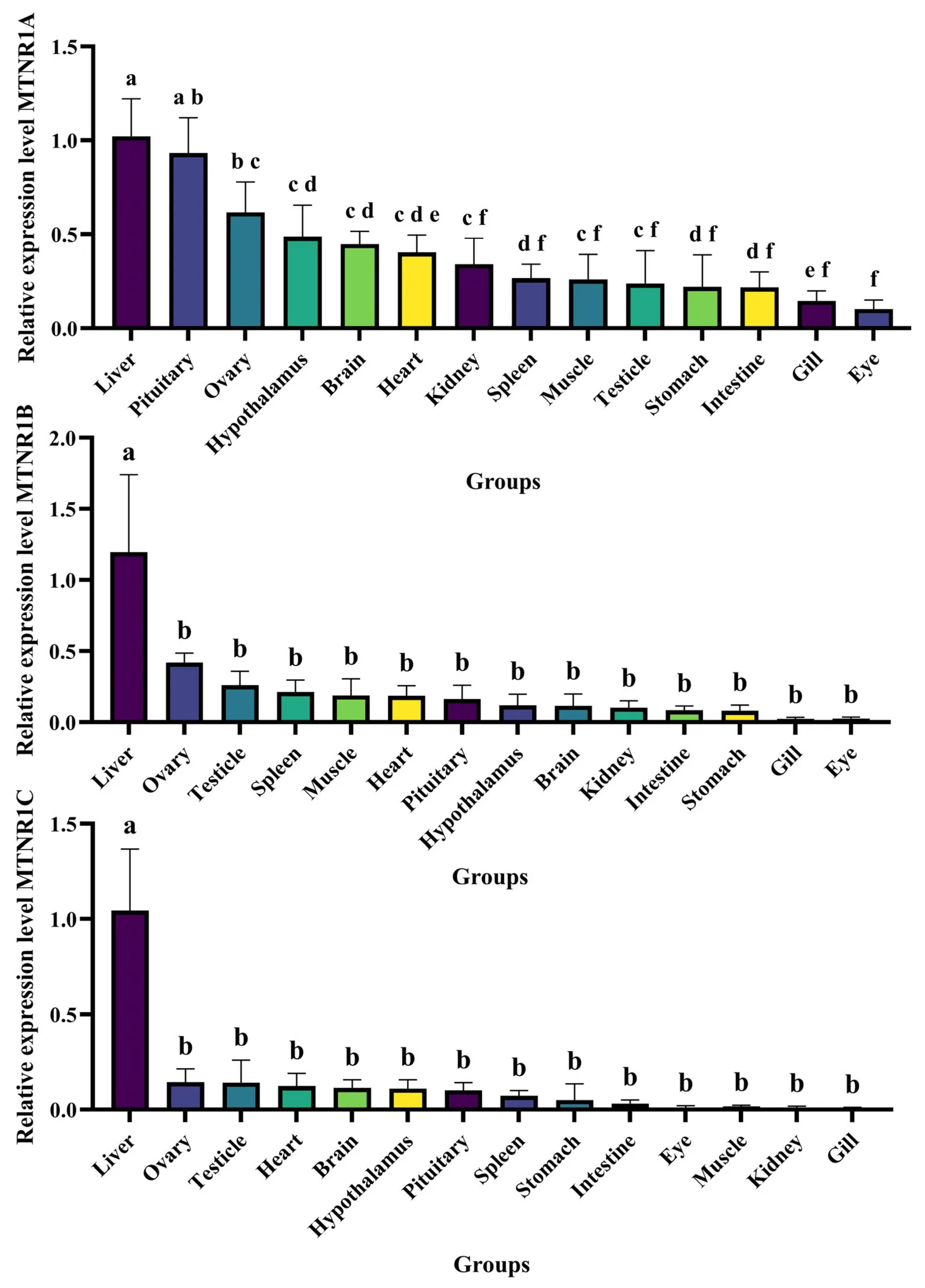
Tissue distribution of *PgMtnr1A*, *PgMtnr1B* and *PgMtnr1C*. RT-qPCR is used to detect the transcription levels in liver, pituitary, kidney, muscle, gill, intestine, eye, brain, spleen, stomach and heart. All results are presented as the fold change relative to the expression in eye after normalized to the expression levels of β-actin. Statistical comparisons among multiple tissues were performed using one-way ANOVA followed by Tukey’s post hoc test. Bars denote the mean ± SEM (*n* = 6). Different letters above error bars indicate significant differences (*p* < 0.05).

The expression of *PgMtnr1a, PgMtnr1b,* and *PgMtnr1c* mRNA was detectable in the tissues of healthy fish. *PgMtnr1a* exhibited the highest expression levels in the liver, pituitary, ovary, and hypothalamus, followed by the brain, heart, kidney, spleen, muscle, testis, stomach, intestine, gill, and eye. This pattern suggests its involvement in energy metabolism, photoperiodic regulation, and reproduction (Figure 5). Real-time PCR results indicated that *PgMtnr1b* mRNA was predominantly expressed in the liver, with lower levels observed in the ovary, testis, spleen, muscle, heart, and pituitary of *P. glenii* (Figure 5). Furthermore, qRT-PCR analysis revealed that *PgMtnr1c* expression was highest in the liver of *P. glenii*, followed by the ovary and testis, with relatively low levels detected in the heart, brain, hypothalamus, and pituitary.

### 3.4. PgMtnr1a, PgMtnr1b, and PgMtnr1c mRNA Expression During Recovery After Freezing

**Figure 6:**
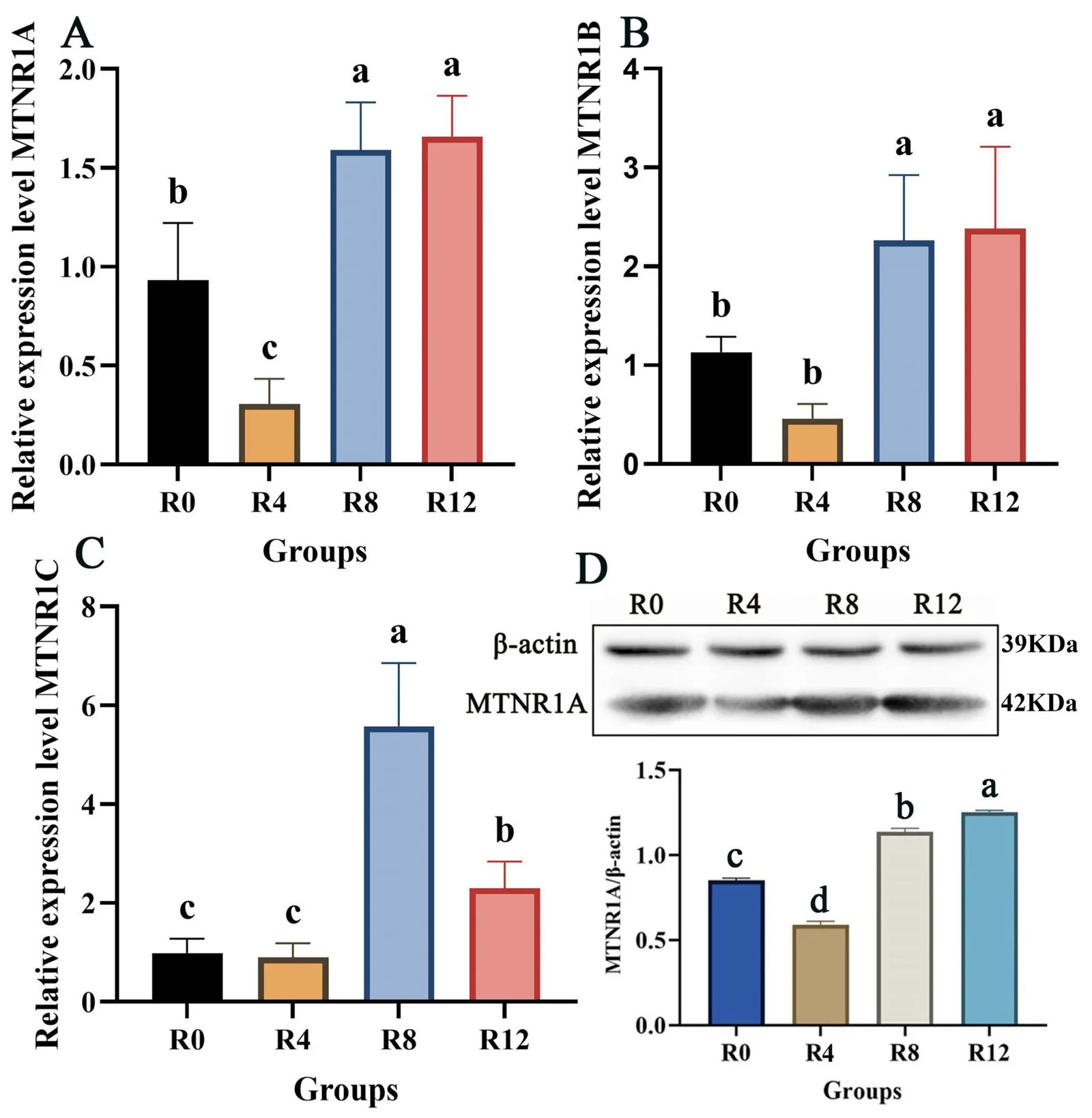
*PgMtnr1A*, *PgMtnr1B* and *PgMtnr1C* expression in the liver during recovery (0 h, 4 h, 8 h and 12 h) after freezing. RT-qPCR is used to detect the *PgMtnr1a* (**A**), *PgMtnr1b* (**B**) and *PgMtnr1c* (**C**) transcription levels. All results present as the fold change relative to the control group after normalized to the expression levels of β-actin. Western blotting analysis of PgMTNR1A in the liver at 0, 4, 8, and 12 h of recovery after freezing (**D**). Statistical comparisons among multiple tissues were performed using one-way ANOVA fol-lowed by Tukey’s post hoc test. Bars denote the mean ± SEM (*n* ≥ 6). Different letters above error bars indicate significant differences (*p* < 0.05).

Dynamic expression changes of *PgMtnr1a*, *PgMtnr1b*, and *PgMtnr1c* in the liver were observed during the recovery period following freezing (Figure 6). The results indicated significant alterations in the gene expression levels of *PgMtnr1a*, *PgMtnr1b*, and *PgMtnr1c* in the liver during the recovery phase from 0 h to 12 h. In addition, the *PgMtnr1a* levels exhibited a rapid decrease followed by an increase, peaking in the R12 group. Notably, significant differences in *PgMtnr1b* gene expression were observed in the R8 and R12 groups compared to the baseline at R0 group (*p* < 0.05, Figure 6). Furthermore, *PgMtnr1c* expression was markedly upregulated in the R8 group (*p* < 0.05) and gradually decreased in the R12 group (*p* < 0.05). Moreover, we examined PgMTNR1A protein expression by Western blot, and the protein levels exhibited a similar dynamic pattern to its mRNA during the recovery period.

### 3.5. Receptor-Dependent Antioxidant Effects of Melatonin

**Figure 7:**
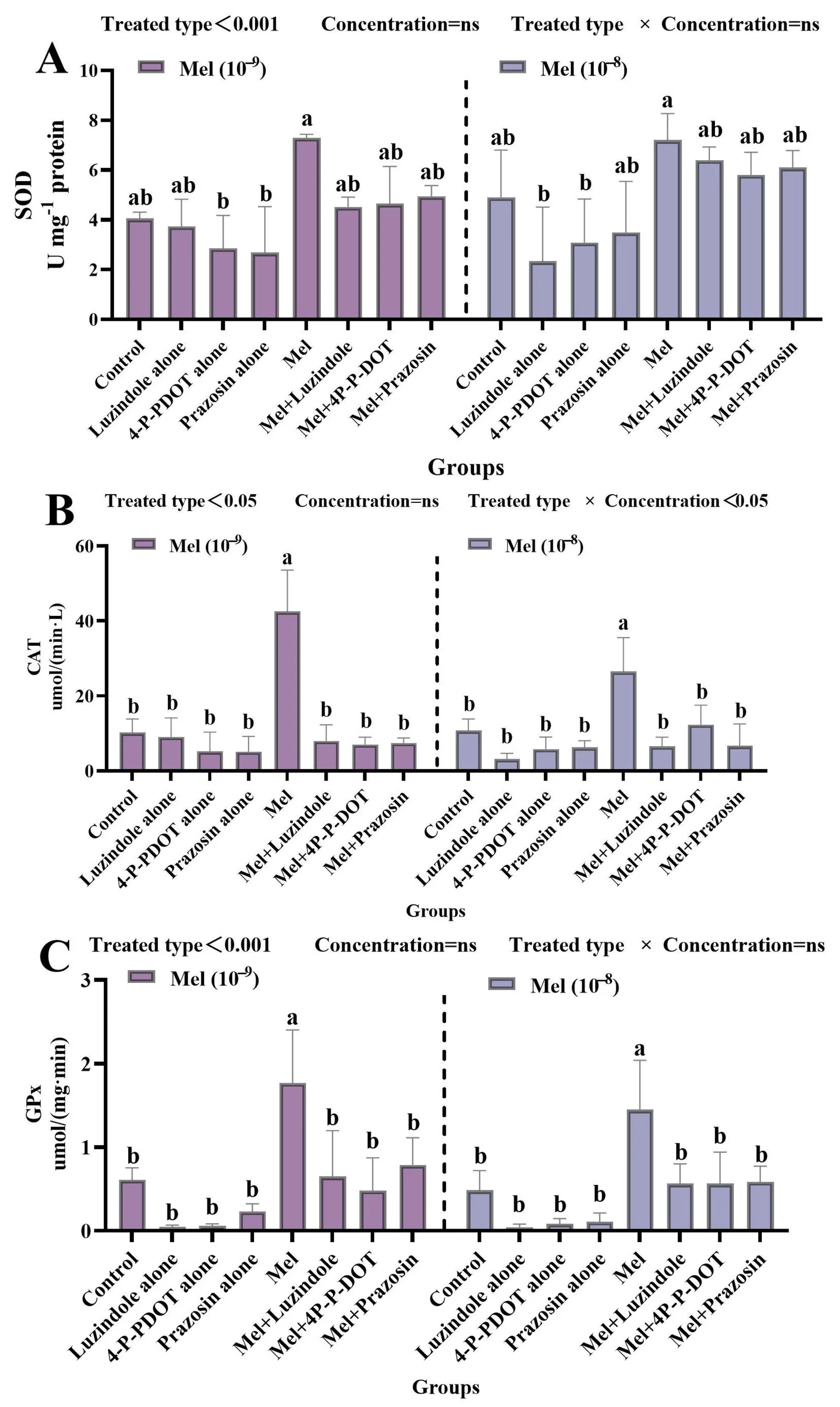
The roles of melatonin receptors according to the effects of melatonin (10^−9^ M, 10^−8^ M), and melatonin receptor antagonists (luzindole, 4P-P-DOT, and prazosin, 10^−7^ M, 10^−6^ M) on the (**A**) superoxide dismutase (SOD), (**B**) catalase (CAT), and (**C**) glutathione peroxidase (GPx) level in the liver tissue in vitro. *n* = 6 for each group. Statistical comparisons among multiple tissues were performed using two-way ANOVA. Different letters indicate significant differences between treated groups.

The effects of melatonin receptors on antioxidant enzyme activity in liver tissue were investigated by assessing the activities of superoxide dismutase (SOD), catalase (CAT), and glutathione peroxidase (GPx) (Figure 7). In both the 10^−9^ and 10^−8^ M groups, treatment with luzindole alone, 4P-P-DOT alone, or prazosin alone did not significantly alter SOD, CAT, or GPx activities compared to the control group (*p* > 0.05), indicating that these antagonists alone had no inherent effect on baseline antioxidant enzyme activity. In the 10^−9^ and 10^−8^ M Mel group, the SOD activity in the melatonin treatment group was the highest, and the differences among the groups were influenced by the treatment type (*p* < 0.05, Figure 7). In the melatonin + antagonist combination groups, SOD activity was significantly lower than that in the melatonin alone group (*p* < 0.05), but did not differ from the respective antagonist-only groups. In the 10^−9^ and 10^−8^ M Mel group, the CAT and GPx activity in the melatonin treatment group was significantly higher than that of the control and all antagonist-only groups (*p* < 0.05, Figure 7), while the activities of each antagonist-only treatment group remained low and similar to control. Treatment type exerted a highly significant effect on SOD activity (*p* < 0.05), with no interaction observed between concentration. Also, there was a highly significant main effect of treatment on GPx activity (*p* < 0.0001), with no interaction observed between concentration. Collectively, these data indicate that the antioxidant effects of melatonin are mediated, at least in part, through melatonin receptor-dependent pathways, as the antagonists effectively attenuated melatonin-stimulated enzyme activities.

### 3.6. Melatonin Receptors Mediate Anti-Inflammatory Responses

**Figure 8:**
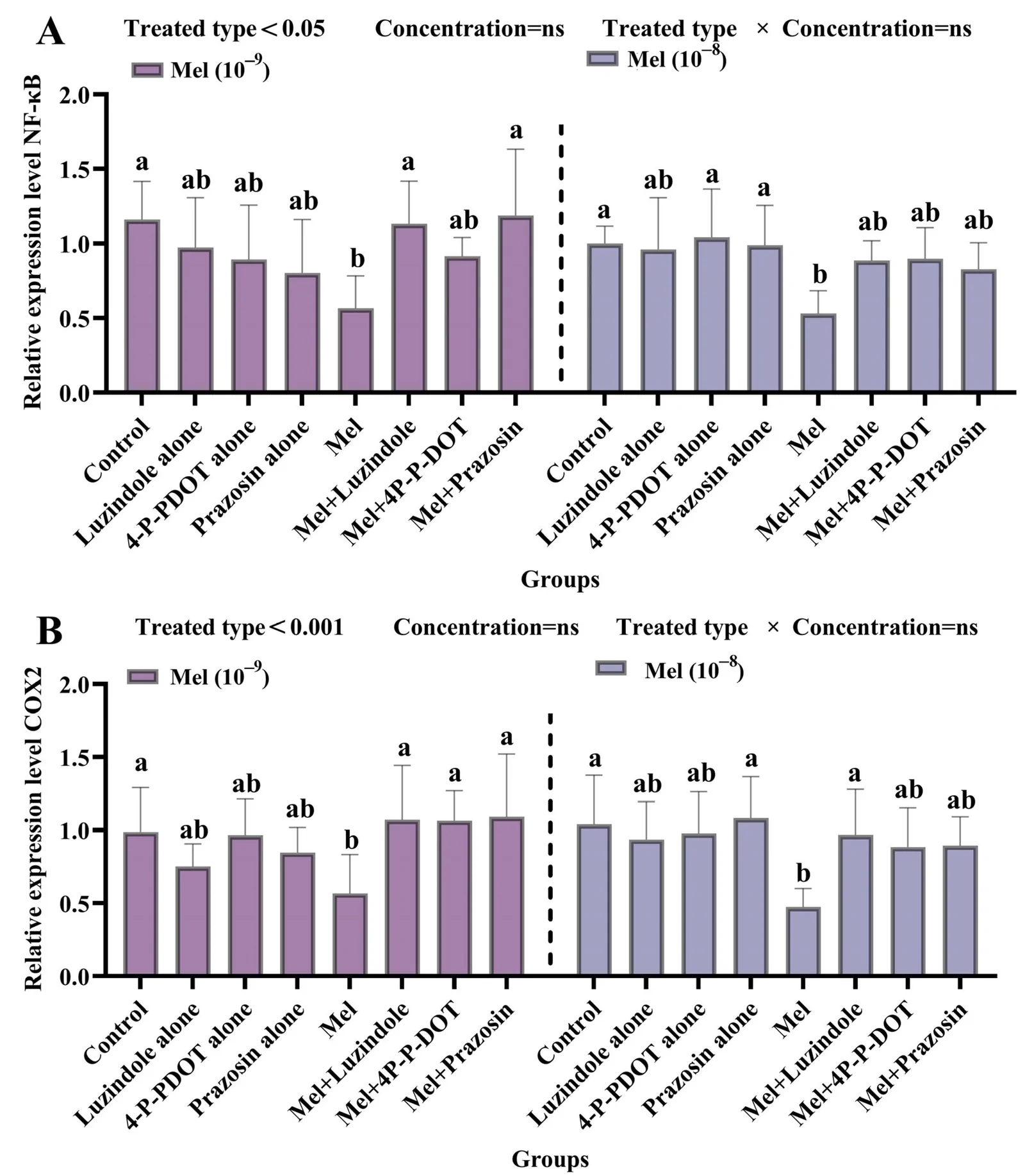
The roles of melatonin receptors according to the effects of melatonin (10^−9^ M, 10^−8^ M), and melatonin receptor antagonists (luzindole, 4P-P-DOT, and prazosin, 10^−7^ M, 10^−6^ M) on the mRNA expression of *Nf-κb* and *Cox2* in *P. glenii* hepatocytes, also in the presence of melatonin. *n* ≥ 6 for each group. Different letters indicate significant differences between treated groups. Statistical comparisons among multiple tissues were performed using two-way ANOVA. Different letters indicate significant differences between treated groups. (**A**) qPCR analysis of *Nf-κb* mRNA levels; (**B**) *Cox2*.

The effects of melatonin receptors on immunology in hepatocytes were investigated by assessing the expression levels of immune-related genes (*Nf-κb* and *Cox2*) using quantitative reverse transcription polymerase chain reaction (qRT-PCR) (Figure 8). The melatonin concentrations used (10^−9^ and 10^−8^ M) were selected based on preliminary dose–response experiments in *P. glenii* hepatocytes, as well as on our measurement of endogenous melatonin levels in this species, which were found to be approximately 10^−9^ M. The concentration of 10^−8^ is consistent with previous reports in other fish species, where effective melatonin doses often range from 10^−8^ to 10^−6^ M due to species-specific differences in metabolic rate and receptor sensitivity. Compared with the blank control group, treatment with Mel alone (10^−9^ M and 10^−8^ M) significantly reduced the mRNA levels of *Nf-κb* and *Cox2* (*p* < 0.05 or *p* < 0.001). Treatment with luzindole, 4P-P-DOT, or prazosin alone (10^−7^ and 10^−6^ M) resulted in no significant changes or only slight increases in the mRNA expression levels of *Nf-κb* and *Cox2* relative to the control group (*p* > 0.05, Figure 8), indicating that the antagonists themselves do not induce obvious inflammation. In the combined treatment groups (Mel + antagonist), the levels of *Nf-κb* and *Cox2* showed no significant difference from the control group but were higher than those in the Mel-only groups (10^−9^ M and 10^−8^ M). These findings suggest that melatonin requires functional melatonin receptors to inhibit the mRNA expression of *Nf-κb* and *Cox2*. The treated type had an extremely significant impact on *Nf-κb* mRNA expression (*p* < 0.05), with no interaction observed between concentration. The treated type had an extremely significant impact on *Cox2* mRNA expression (*p* < 0.001), with no interaction observed between concentration. We also examined the protein expression of Nf-κb and *Cox2* by Western blot (Figure S1). Consistent with the mRNA trends, melatonin treatment alone significantly reduced the expression of both proteins. In the Mel + Luzindole and Mel + 4P-P-DOT groups, COX2 protein expression was markedly restored. For NF-κB, however, the changes in protein levels in the combined treatment groups did not fully correspond to those observed at the mRNA level, as the receptor antagonists failed to restore NF-κB protein expression, possibly reflecting slower protein turnover.

### 3.7. Effects of MTNR1A Overexpression on Related Gene Expression

**Figure 9:**
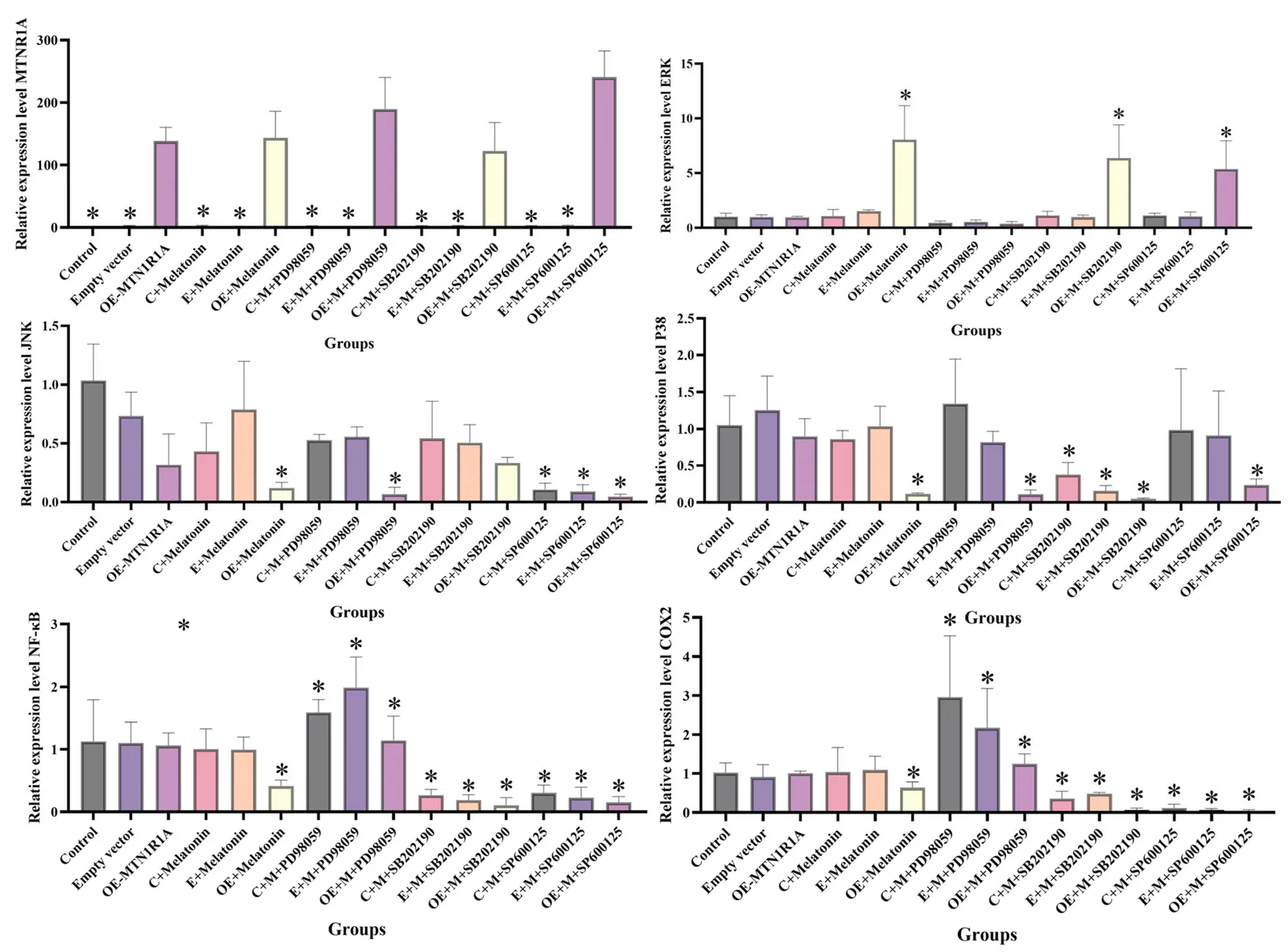
Relative expression levels of genes (*Mtnr1a*, *Erk*, *p38*, *Cox2*, *Jnk* and *Nf-κb*) in *PgMtnr1a* overexpression and HEK293 cell experiments. Primary hepatocytes were transfected and treated with melatonin and pathway inhibitors. RT-qPCR was used to detect gene transcription levels, normalized to β-actin. Results are presented as fold changes relative to the control group. Bars denote the mean ± SEM (*n* = 3 biological replicates). * indicates that the expression level was significant compared with the OE-MTNR1A group (*p* < 0.05). Statistical comparisons among multiple tissues were performed using one-way ANOVA followed by Tukey’s post hoc test.

To explore the regulatory mechanism of melatonin on the MAPK signaling pathway (*Erk*, *Jnk*, *p38*) and downstream inflammatory factors (*Nf-κb* and *Cox2*) through its receptor MTNR1A, we conducted a combined experiment involving the overexpression of MTNR1A, the administration of melatonin, and multiple pathway inhibitors (Figure 9). In the control and empty vector groups, the expression level of the *ERK* gene did not show a significant difference compared to the OE-MTNR1A group. Similarly, in the C + Melatonin and E + Melatonin groups, the expression level of the *ERK* gene was comparable to that in the OE-MTNR1A group. However, in the OE + Melatonin group, the expression level of the *ERK* gene increased significantly, demonstrating a notable rise compared to the OE-MTNR1A group. In the C + M + PD98059, E + M + PD98059, and OE + M + PD98059 groups, the expression level of the *ERK* gene did not differ significantly from that in the OE-MTNR1A group. These results indicate that MTNR1A overexpression, in conjunction with melatonin, leads to a significant upregulation of ERK mRNA expression, and this effect can be reversed by the ERK inhibitor PD98059. In groups treated with melatonin alone or with melatonin receptor overexpression (OE-MTNR1A), the expression level of the *Jnk* gene decreased, with a significant reduction observed in the OE + Melatonin group. In all groups treated with the JNK signaling pathway inhibitor (C + M + SP600125, E + M + SP600125, OE + M + SP600125), the expression levels were significantly lower than those in the OE-MTNR1A group. The results indicate that melatonin treatment, particularly in the presence of MTNR1A overexpression, is associated with a significant downregulation of *Jnk* mRNA expression, an effect that is attenuated by the JNK inhibitor SP600125. In the OE + Melatonin group, the expression level of the *p38* gene was significantly lower than that in the OE-MTNR1A group. Furthermore, in the C + M + SB202190, E + M + SB202190, and OE + M + SB202190 groups, the expression levels of the *p38* gene were significantly lower than those in the OE-MTNR1A group. Additionally, *p38* gene expression in the OE + M + PD98059 group was significantly lower than that in the OE-MTNR1A group, and similarly, *p38* gene expression in the OE + M + SP600125 group was significantly lower than that in the OE-MTNR1A group. These findings suggest that melatonin receptor overexpression, when combined with the p38 inhibitor SB202190, leads to a further significant reduction in *p38* mRNA expression, indicating a potential synergistic effect at the transcriptional level. The expression levels of the *Nf-κb* and *Cox2* genes in the Control and Empty vector groups were comparable to those in the OE-MTNR1A group, indicating no significant difference in *Nf-κb* and *Cox2* expression under basal conditions compared to the receptor overexpression group. Notably, the *NF-κB* and *COX2* gene expression levels in the OE + Melatonin group were lower than those in the OE-MTNR1A group, demonstrating that melatonin combined with receptor overexpression synergistically reduces the mRNA expression levels of *NF-κB* and *Cox2*. In the E + M + PD98059 and OE + M + PD98059 groups, the *Nf-κb* and *Cox2* gene expression levels were significantly higher than those in the OE-MTNR1A group. Conversely, in the C + M + SB202190, E + M + SB202190, OE + M + SB202190, E + M + SP600125, and OE + M + SP600125 groups, the expression levels of *Nf-κb* and *Cox2* were significantly lower than those in the OE-MTNR1A group. The OE-MTNR1A group did not exhibit a significant effect on the expression of *Nf-κb* and *Cox2*. However, when combined with melatonin or specific inhibitors such as SB202190 and SP600125, there was a significant reduction in the expression levels of *Nf-κb* and *Cox2*. Conversely, the combination with PD98059 resulted in a significant increase in the expression of *NF-κB* and *COX2*. Importantly, the regulatory trends of MAPK components and inflammatory genes observed in HEK293T cells with human-specific primers were consistent with those obtained from *P. glenii* primary hepatocytes using fish-specific primers (Figure S2), confirming receptor-dependent signaling.

## 4. Discussion

**Figure 10:**
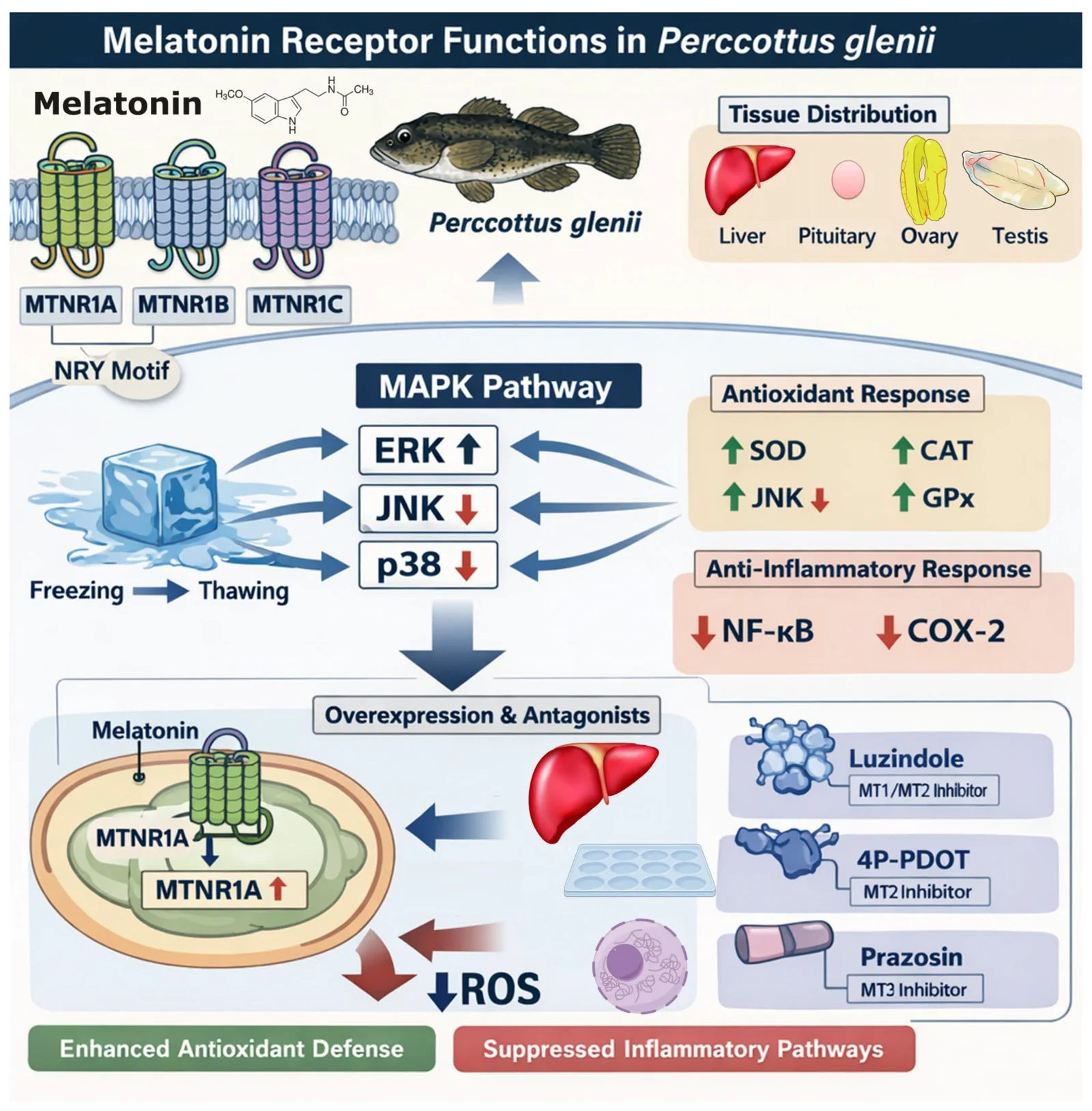
The antioxidant and anti-inflammatory mechanisms mediated by melatonin receptors.

A conceptual model integrating the key findings of the present study is presented in Figure 10. To the best of our knowledge, this study presents the first comprehensive characterization of *Mtnr1a*, *Mtnr1b*, and *Mtnr1c* in *P. glenii*, encompassing nucleotide and protein sequences, hepatic expression patterns, and functional roles during post-freezing recovery. In zebrafish, a single copy of mtnr1aa, mtnr1ab, mtnr1bb, and mtnr1ba was identified, while in the Kanglang white minnow, grass carp, Amur ide (*Leuciscus waleckii*), and three Chinese golden-line barbels (*Sinocyclocheilus anshuiensis*, *S. graham*, and *S. rhinocerous*), two copies of mtnr1a (mtnr1aa and mtnr1ab) and two copies of mtnr1b (mtnr1ba and mtnr1bb) were recognized [46]. In contrast, the majority of tetrapods exhibited only a single copy of both mtnr1a and mtnr1b, whereas fish presented one copy of mtnr1c. This investigation marks the first cloning of complete cDNA encoding a functional melatonin receptor from the liver of *P. glenii*. We successfully cloned three subtypes of melatonin receptors in *P. glenii* to elucidate the mechanisms through which melatonin and its receptors facilitate recovery from freezing. The melatonin receptors exhibited seven transmembrane domains, a characteristic feature of the GPCR family, interconnected by a series of intracellular and extracellular loops [47,48]. Essential amino acid motifs pertinent to these functions were found to be conserved across fish species [49,50,51]. Notably, these motifs include two serine residues in transmembrane domain III, two cysteine residues in the fourth loop domain, valine and histidine residues in transmembrane domain IV, and a proline along with a serine residue in transmembrane domains VI and VII. Recent research has demonstrated that MTNR1A exhibits the highest affinity for melatonin [52], whereas MTNR1C shows the lowest affinity among the three primary melatonin receptor subtypes [47]. In true mammals, MTNR1C appears to have lost its capacity to bind melatonin, in contrast to the functional Mel1c present in fish, thereby underscoring the uniqueness and significance of this receptor in non-mammalian species.

To further investigate the functional roles of melatonin receptors in *P. glenii*, this study examined their tissue distribution at the mRNA level using quantitative PCR (qPCR). The results revealed widespread expression of *PgMtnr1a*, *PgMtnr1b* and *PgMtnr1c* across multiple tissues, with particularly high transcript levels detected in the liver, pituitary, ovary, and testis. These findings suggest a potential involvement of melatonin signaling in light-related regulation, reproduction, and energy metabolism in *P. glenii*, warranting further functional validation. The observed mRNA distribution pattern aligns with reports from several other teleost species. In *Solea senegalensis*, *Mtnr1a* expression was also prominent in the liver [27], while in the mudskipper *Boleophthalmus pectinirostris*, *Mtnr1a* exhibited high expression in the brain and ovary [19]. Similarly, studies in *Dicentrarchus labrax* and *Esox lucius* indicated strong melatonin receptor expression in the optic tectum, retina, and pituitary [19,53]. Consistent with these observations, multiple studies across different vertebrate species have detected melatonin receptor expression in ovarian tissues, supporting the conserved role of melatonin in reproductive regulation [30,54,55,56]. In the present study, *PgMtnr1a* exhibited not only high expression in the liver but also clear mRNA signals in peripheral tissues including the spleen, kidney, heart, and muscle, whereas the expression of *PgMtnr1b* and *PgMtnr1c* in these peripheral tissues was relatively limited. Given the central role of the liver in systemic metabolism and the predominant expression of PgMTNR1A in this organ, the selection of this receptor for functional overexpression validation represents an optimal matching strategy based on receptor abundance and tissue specificity. The rationale for prioritizing MTNR1A in the present study is reinforced by previous findings in *D. labrax* and *S. senegalensis* [19,27]. It is important to note, however, that the present study reflects mRNA expression patterns, which may not always correspond directly to protein abundance or functional receptor localization. Furthermore, comparative analysis highlights significant interspecific variation in melatonin receptor distribution among fish species, suggesting possible species-specific adaptations in melatonin-mediated physiological regulation. Future studies incorporating protein-level detection would help to clarify the functional localization of these receptors in *P. glenii*.

During the recovery from freezing in *P. glenii*, the expression of all three receptor subtypes was dynamically regulated. Notably, the expression of *PgMtnr1b* and *PgMtnr1c* was significantly upregulated at R8, while *PgMtnr1a* expression peaked at R12. The recovery process from freezing resembles ischemia–reperfusion [57]; however, it remains unclear whether melatonin exerts a protective role through its receptors during ischemia–reperfusion. Following ischemia–reperfusion injury (IRI), *MTNR1B* is upregulated, suggesting that it may play a crucial role in the cellular response to ischemia–reperfusion injury and may be involved in protective mechanisms, such as activating related signaling pathways by binding with melatonin, alleviating myocardial cell injury and apoptosis, and improving cardiac function [58]. In the present study, the increased mRNA expression levels of *PgMtnr1a*, *PgMtnr1b* and *PgMtnr1c* observed at R8 and R12 suggest that melatonin may directly influence the late recovery period following freezing. We found that the expression levels of *Cox2* and *Nf-κb* were significantly higher following luzindole treatment compared to melatonin (MT) treatment. Notably, existing research indicates that luzindole may exert biological effects independent of MT receptors in mammalian models. This observation suggests that luzindole’s role may extend beyond merely antagonizing the MT pathway, implicating potential off-target effects as well [59,60]. Some studies have reported that luzindole can act as an inverse agonist or partial agonist at melatonin receptors and may exert effects independent of endogenous melatonin when administered alone [61,62]. To test this hypothesis in our study, we included a single-agent control group (luzindole alone treatment), which partially controls for this potential effect. This study demonstrates that luzindole, a non-selective antagonist, in conjunction with 4-P-PDOT, a selective antagonist targeting MTNR1B, can attenuate the inhibitory effects of melatonin on the expression levels of inflammatory cytokines *Cox2* and *Nf-κb* at the mRNA level. In comparing the effects of melatonin and 4-P-PDOT, the combination of melatonin and luzindole exhibited a significantly reduced inhibitory effect on the expression of inflammatory cytokines, with the diminished effect being more pronounced than that observed with melatonin alone. These findings are consistent with research conducted on sheep epididymal epithelial cells exposed to melatonin in vitro, supporting the assertion that both MTNR1A and MTNR1B are involved in mediating melatonin’s anti-inflammatory actions [63]. Our findings imply that MTNR1A and MTNR1B play crucial roles in facilitating the anti-inflammatory properties of melatonin. Compared to the MT2-selective 4-P-PDOT, the broader and more pronounced effect of the non-selective antagonist luzindole suggests either that the anti-inflammatory actions of melatonin involve coordinated signaling through both MTNR1A and MTNR1B receptors in this model, or that luzindole’s blockade of both subtypes yields a more comprehensive inhibition of melatonin’s effects. Previous studies have confirmed that various fish species, including the cold-tolerant *Takifugu fasciatus*, *T. obscurus*, and *Cyprinus carpio*, generally mitigate tissue inflammatory damage caused by cold temperatures. This is achieved by upregulating the expression of anti-inflammatory related genes and enhancing the activity of anti-inflammatory pathways in response to low temperatures, thereby facilitating adaptation to cold environments [64,65,66]. Teleosts exhibit distinct melatonin physiology and receptor sensitivity compared with mammals effective concentrations in fish often range from 10^−8^ to 10^−6^ M, which are considered pharmacological but relevant in cellular models of stress resistance [67,68]. Luzindole (*N*-acetyl-2-benzyltryptamine) acts as a competitive antagonist of melatonin receptors and has been extensively used in research to investigate melatonin cellular effects, broadly inhibiting both MT1 (MTNR1A) and MT2 (MTNR1B) receptor subtypes [69,70,71]. 4-P-PDOT is reported as a specific MT2 inhibitor, and prazosin is a selective MT3 melatonin receptor antagonist in several vertebrate systems [72,73]. In teleosts, pharmacological evidence from *Labeo rohita* and *Channa punctatus* has supported the use of prazosin as an MT_3_-selective antagonist [74,75]. However, given that the pharmacological profiles of these antagonists may vary across species, receptor paralogs, and cell types, their specificity in *P. glenii* awaits further validation. Nevertheless, the consistent blockade of melatonin-induced effects by these antagonists across multiple fish species, combined with our MTNR1A overexpression data, is consistent with the interpretation that MTNR1A contributes to mediating the observed responses in *P. glenii*. Enzymes such as SOD, GPx, and CAT are known to be involved in antioxidant defense [55]. In bovine oocytes, it was confirmed that melatonin upregulated the expression of CAT, SOD and GPx [76]. In vitro experiments have shown that melatonin can exert antioxidant effects by activating MTNR1B and upregulating the expression of antioxidant enzymes such as SOD2 (superoxide dismutase 2), GPx1 (glutathione peroxidase 1), and HO-1 (heme oxygenase 1), further inhibiting liver fibrosis [77]. The results of this study further confirmed in the liver cells of the *P. glenii* that the regulation of melatonin against oxidase activity is dose-dependent and partially dependent on its receptor pathway. Treatment with melatonin alone significantly enhanced the activities of SOD, CAT and GPx. Studies in bovine ovarian follicles have confirmed that there is a receptor-mediated regulatory relationship between MTNR1A/B and antioxidant enzymes (SOD, CAT). Melatonin needs to bind to the MTNR1A/B receptor in ovarian follicles to initiate the regulation of antioxidant enzyme gene expression. Once the receptor is blocked by luzindole, its upregulation effect on antioxidase will be significantly weakened or even disappear [55]. In this study, when the broad-spectrum receptor antagonist luzindole was added, this boosting effect was significantly inhibited, especially at a concentration of 10^−8^ M Mel, which directly supported the key role of the receptor-mediated mechanism in this process.

Building upon the antagonist studies, our *PgMtnr1a* overexpression experiments further clarified its specific role. The study confirmed through *PgMtnr1a* overexpression combined with melatonin treatment that MTNR1A serves as a critical regulator of inflammatory responses by coordinately modulating the MAPK/NF-κB signaling cascade. Experimental results demonstrated that *PgMtnr1a* overexpression significantly suppressed the NF-κB/COX2 inflammatory pathway at the transcriptional level. This suppression aligns with the antagonist data showing luzindole (affecting MTNR1A) inhibited high-concentration melatonin-induced NF-κB upregulation. Although the differential molecular mechanisms have not been discussed, existing work has clearly involved and reported the differential effects of different melatonin concentrations in improving obsessive–compulsive disorder-like symptoms in mammals, confirming that melatonin treatment has a dose-dependent effect [78]. The observed downregulation of *Cox2* and *Nf-κb* mRNA expression suggests that the MT1 receptor may exert anti-inflammatory effects through multiple mechanisms including SIRT1 activation and Gi/o-IKK pathway regulation [79,80]. These findings align with previous studies demonstrating melatonin-mediated suppression of NF-κB activation via MT1 receptors [81]. Furthermore, our results showing antagonist-mediated reduction of *COX2* expression under both low and high-melatonin conditions suggest a complex, concentration-dependent regulatory role for melatonin receptors on *COX2*, which is consistent with the overarching anti-inflammatory role of MTNR1A activation identified in the overexpression model. The differential effects of receptor antagonists under low versus high melatonin concentrations suggest a complex, concentration-dependent regulatory relationship. While the precise mechanism requires further study, our data are consistent with a model demonstrating that receptor availability or coupling efficiency may be modulated by ligand concentration, potentially involving processes such as receptor desensitization or negative feedback. Studies have shown that melatonin downregulated the expression or density of its receptors, such as the MT2 receptor in the suprachiasmatic nucleus, following pretreatment with physiological concentrations [82,83]. This autoregulation creates an approximate inverse correlation between endogenous melatonin levels and receptor availability. Consequently, under lower exogenous melatonin conditions, the system might be more sensitive to the disruptive effect of the antagonist (luzindole) on receptor homeostasis, leading to a more pronounced downregulation of receptor gene expression. In contrast, at higher melatonin concentrations, the receptor pool may already be substantially suppressed or saturated, rendering the additional impact of the antagonist less dramatic.

In addition, compared with the control group, the expression of the *ERK* gene was significantly increased, while the expressions of the *p38* and *JNK* genes were significantly decreased in the OE-MTNR1A + Melatonin group (Figure 8). Furthermore, our data suggest that MTNR1A may modulate inflammatory responses, at least in part, through differential regulation of MAPK pathway transcription. We observed that *PgMtnr1a* overexpression, in the presence of melatonin, was associated with decreased mRNA expression of *JNK* and *p38*, and increased mRNA expression of ERK, consistent with a potential regulatory role via mechanisms such as Gi/o-PI3K [79]. Future studies investigating the phosphorylation status of these proteins would further elucidate the downstream signaling mechanisms at the post-translational level. This coordinated regulation may involve ROS suppression and inhibition of upstream kinases such as ASK1 [84]. Consequently, these transcriptional changes in MAPK pathway components were associated with the suppression of the downstream NF-κB/COX2 inflammatory axis, aligning with the anti-inflammatory role identified in the antagonist model. Notably, melatonin treatment enhanced these effects, indicating synergistic action between endogenous receptor activation and exogenous ligand stimulation. Meanwhile, pathway inhibitor experiments revealed complementary mechanisms between MTNR1A signaling and MAPK inhibitors. While MTNR1A primarily targets ROS-dependent pathways, JNK/p38 inhibitors directly block kinase activity, with combined application producing enhanced anti-inflammatory effects [40]. Conversely, *ERK* inhibition significantly attenuated MTNR1A-mediated anti-inflammatory responses, confirming ERK’s crucial role as a downstream effector of melatonin signaling [81]. The identification of the MTNR1A-MAPK/NF-κB axis as a key pathway in our study resonates with the genomic findings of Jiang et al. (2023), who reported that genes involved in neuroprotective adaptations, including those related to neurotransmission and cellular stress, are under positive selection in *P. glenii*, suggesting that multiple signaling pathways have evolved to coordinate survival during freezing and recovery [36]. Collectively, our receptor-dependency studies, incorporating both pharmacological antagonism and PgMtnr1a overexpression, support MTNR1A as a key mediator of melatonin’s immunomodulatory and antioxidant effects in *P. glenii* liver. The markedly enhanced response in PgMtnr1a-overexpressing cells compared to melatonin treatment alone, along with evidence from MTNR1A -KO models [80], consolidates this receptor’s central role in transducing melatonin’s bioactivity. Our receptor-dependency studies, incorporating both pharmacological antagonism and genetic overexpression, unequivocally established MTNR1A as a primary mediator of melatonin’s immunomodulatory effects. The markedly enhanced response in *PgMtnr1a*-overexpressing cells compared to melatonin treatment alone, along with the specific inhibitory effects of luzindole on melatonin-induced signaling, consolidates this receptor’s central role in transducing melatonin’s bioactivity. These findings extend current understanding of melatonin receptor function to fish models, providing important insights into the evolutionary conservation of melatonin receptor functions (Figure 9). It is noteworthy that the receptor-dependent mechanisms elucidated here represent one major mode of melatonin action. In certain biological contexts, such as cat oocytes, melatonin receptors have not been identified (receptor level is extremely low) despite their presence in other species [85,86,87]. This scenario highlights the receptor-independent capacity of melatonin, attributable to its small molecular size and lipophilic nature, which enables direct membrane diffusion and intracellular antioxidant activity even in the absence of canonical receptors (low receptor level) [86,87]. This dual capacity, both receptor-mediated signaling and direct non-receptor actions, underscores the functional versatility of melatonin across vertebrates. Our study in *P. glenii* now clearly establishes the critical role of the receptor-dependent pathway, specifically via MTNR1A, in coordinating the antioxidant and anti-inflammatory responses during a physiologically extreme event like freeze recovery. We observed that melatonin induced its own receptor mRNA expression, and this effect was partially blocked by receptor antagonists. However, we did not include a protein synthesis inhibitor to confirm that the increase in receptor mRNA requires de novo protein synthesis, nor did we measure melatonin receptor protein levels. Therefore, the correlation between mRNA expression and functional receptor protein remains to be established. Furthermore, this study lacks direct assessments of survival rates, physiological performance, or long-term recovery following freezing. While our molecular data demonstrate that melatonin receptors modulate antioxidant and inflammatory responses during early recovery, how these biochemical changes translate into organismal fitness and freeze tolerance remains unclear. It should be acknowledged that the commercial antibodies used for COX2 and NF-κB Western blot analysis produced consistent non-specific bands despite repeated optimization. Consequently, the conclusions regarding COX2 and NF-κB expression in this study are primarily drawn from qRT-PCR data at the transcriptional level. Future studies should integrate long-term survival monitoring, physiological evaluations, and protein-level analyses to establish the functional and ecological significance of the melatonin receptor system in freeze recovery and to explore whether the MTNR1A-MAPK/NF-κB axis is conserved in other freeze-tolerant vertebrates.

## 5. Conclusions

In conclusion, this study presents the first comprehensive characterization of three melatonin receptor subtypes (*PgMtnr1a*, *PgMtnr1b*, and *PgMtnr1c*) in the freeze-tolerant fish *P. glenii.* These receptors are broadly expressed in metabolically active tissues, with their mRNA expression significantly upregulated during the late phase of post-freezing recovery (R8–R12), indicating that they may contribute to stress repair and homeostasis reconstruction process in the later stage of resuscitation. Functional studies have confirmed that these receptors mediate the core protective effect of melatonin in a receptor-dependent manner. Receptor antagonists can block the activation of the melatonin antioxidant enzyme system and reverse its inhibitory effect on the NF-κB/COX2 inflammatory pathway. Mechanistically, PgMTNR1A is the core regulator. At the mechanistic level, *PgMtnr1a* overexpression experiments identified this receptor subtype as a key mediator of melatonin’s actions. Its overexpression can work in synergy with melatonin to differentially regulate the mRNA expression of MAPK pathway components (upregulating *Erk* while downregulating *Jnk*/*p38*), which is associated with the suppression of the *Nf-κb*/*Cox2* inflammatory axis at the transcriptional level during the late stage of resuscitation. Collectively, these findings support a critical role for melatonin receptor signaling, particularly via PgMTNR1A, in the late phase of freeze–thaw recovery in *P. glenii*, providing new molecular and physiological insights into melatonin’s functions under extreme environmental stress in vertebrates.

## Figures and Tables

**Table 1:** Primers used in cloning and mRNA expression analyses.

Primers	Sequences (5′–3′)	Accession Number	Purpose Used
MTNR1A-F	TACCACAACTTTGGGCTGCTT	PV433192.1	Real-time quantitative PCR
MTNR1A-R	AAATGTTCCCTGCGTTCCTTA		
MTNR1B-F	CAAACCGTCAGCACTTC	PV424064.1	
MTNR1B-R	CACAAATAGCCATTCAGG		
MTNR1C-F	TGCCATCGCCATCAA	PV433193.2	
MTNR1C-R	GCAGTAAGACACCACCAAC		
ERK-F	GTTGCACCGTGACCTCAAAC	SUB16258675	
ERK-R	GAGCATGATCTCTGGGGCTC		
JNK-F	CCTCCAAAGATTCCAGACAAACA	PZ544605	
JNK-R	TTCCATTCCTCCCATTCATACAC		
p38-F	CGTCTTCTCCCCTGCTTC	SUB16367491	
p38-R	CCCCGTCATCTCATCGTC		
NF-κB-F	TTGGGTTTGGGGATTCTA	PZ544606	
NF-κB-R	TTCTGTGTGGGTCTGGTG		
COX2-F	TAGCAGGTAAAAAGGAGC	SUB16367486	
COX2-R	GAAATGTTGAGCGAAGAA		
β-actin-F	CTACTCATTCACCACCACA	SUB16364037	
β-actin-R	GCAACGGAACCTCTCAT		
5CAACGGAACC	TGAGGAGCCAGACCAGAG		RACE
5′-MTNR1B-B	GCAGTAGCGGTTGATGG		
5′-MTNR1B-C	TGAGGAGCCAGACCAGAG		

## Data Availability

The original contributions presented in this study are included in the article and Supplementary Materials. The raw data supporting the conclusions of this article will be made available by the authors on request.

## Supplementary Materials

The following supporting information can be downloaded at: https://www.mdpi.com/article/10.3390/antiox15080978/s1, Figure S1. Elucidate the roles of melatonin receptors by assessing the effects of melatonin, and melatonin receptors antagonists (luzindole, 4P-P-DOT, and prazosin) on the mRNA expression of NF-κB and COX2 in *P. glenii* hepatocytes, also in the presence of melatonin. *n* ≥ 6 for each group. (A) Protein levels in *P. glenii*; (B) Grayscale quantification of NF-κB protein levels in (A); (C) Grayscale quantification of COX2 protein levels in (A). Figure S2. Relative expression levels of genes (*Mtnr1a*, *ERK*, *p38*, *COX2*, *JNK* and *NF-κB*) in *PgMtnr1a* overexpression and HEK293 cell experiments. Human primary hepatocytes were transfected and treated with melatonin and pathway inhibitors. RT-qPCR was used to detect gene transcription levels, normalized to β-actin. Results are presented as fold changes relative to the control group. Bars denote the mean ± SEM (*n* = 3 biological replicates). * indicates that the expression level was significant compared with the OE-MTNR1A group (*p* < 0.05). Statistical comparisons among multiple tissues were performed using one-way ANOVA followed by Tukey’s post-hoc test. Figure S3. Representative Western blot analysis showing the expression of proteins in different experimental groups. (A) β-actin (~42 kDa) for primary cells of *P. glenii*, (B) COX2 (~72 kDa), (C) NF-κB (~65 kDa), (D) β-actin (~65 kDa) for liver tissue of *P. glenii* during recovery from freezing, and (E) MTNR1A (~39 kDa). Protein bands were detected by chemiluminescence and quantified relative to β-actin. Table S1. Primers used in cloning and mRNA expression analyses. Table S2. The accession numbers of all reference sequences used in the phylogenetic analysis.
