# Supplementary material for: Molecular Characterization of Melatonin Receptors in Perccottus glenii and Its Potential Roles in Melatonin-Mediated Antioxidant and Anti-Inflammatory Responses During Post-Freezing Recovery
Source: Antioxidants (Basel). 2026 Aug 6;15(8):978. doi: 10.3390/antiox15080978 (PMC13509318; doi:10.3390/antiox15080978)
Supplement: Supplementary file 1 [file antioxidants-15-00978-s001.zip › antioxidants-4394355-supplementary.pdf]

Fig. S1. Elucidate the roles of melatonin receptors by assessing the effects of melatonin, and melatonin receptors antagonists (luzindole, 4P-P-DOT, and prazosin) on the mRNA expression of NF-κB and COX2 in *P. glenii* hepatocytes, also in the presence of melatonin. n ≥ 6 for each group. (A) Protein levels in *P. glenii*; (B) Grayscale quantification of NF-κB protein levels in (A); (C) Grayscale quantification of COX2 protein levels in (A).

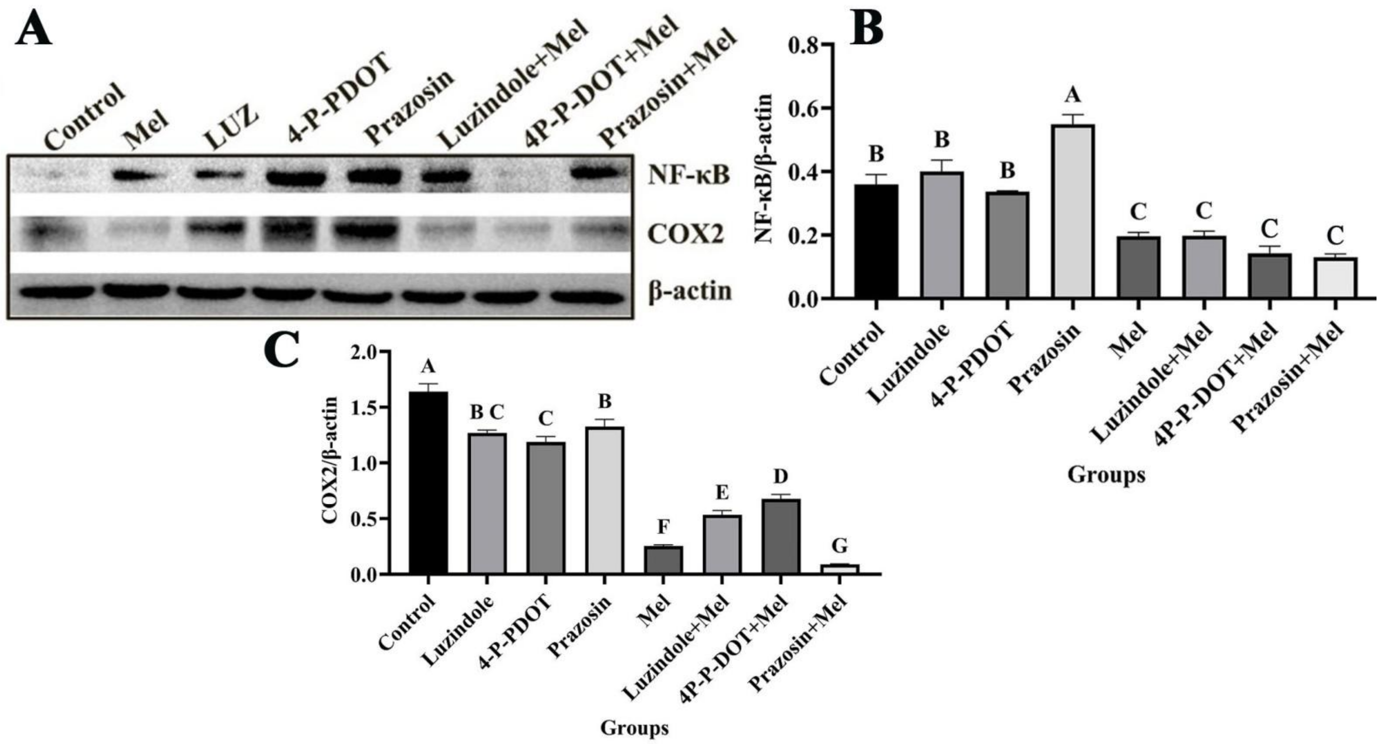

Fig. S2. Relative expression levels of genes (*Mtnr1a*, *ERK*, *p38*, *COX2*, *JNK* and *NF-κB*) in *PgMtnr1a* overexpression and HEK293 cell experiments. Human primary hepatocytes were transfected and treated with melatonin and pathway inhibitors. RT - qPCR was used to detect gene transcription levels, normalized to β- actin. Results are presented as fold changes relative to the control group. Bars denote the mean ± SEM (n = 3 biological replicates). * indicates that the expression level was significant compared with the OE-MTNR1A group ($p < 0.05$). Statistical comparisons among multiple tissues were performed using one-way ANOVA followed by Tukey's post-hoc test.

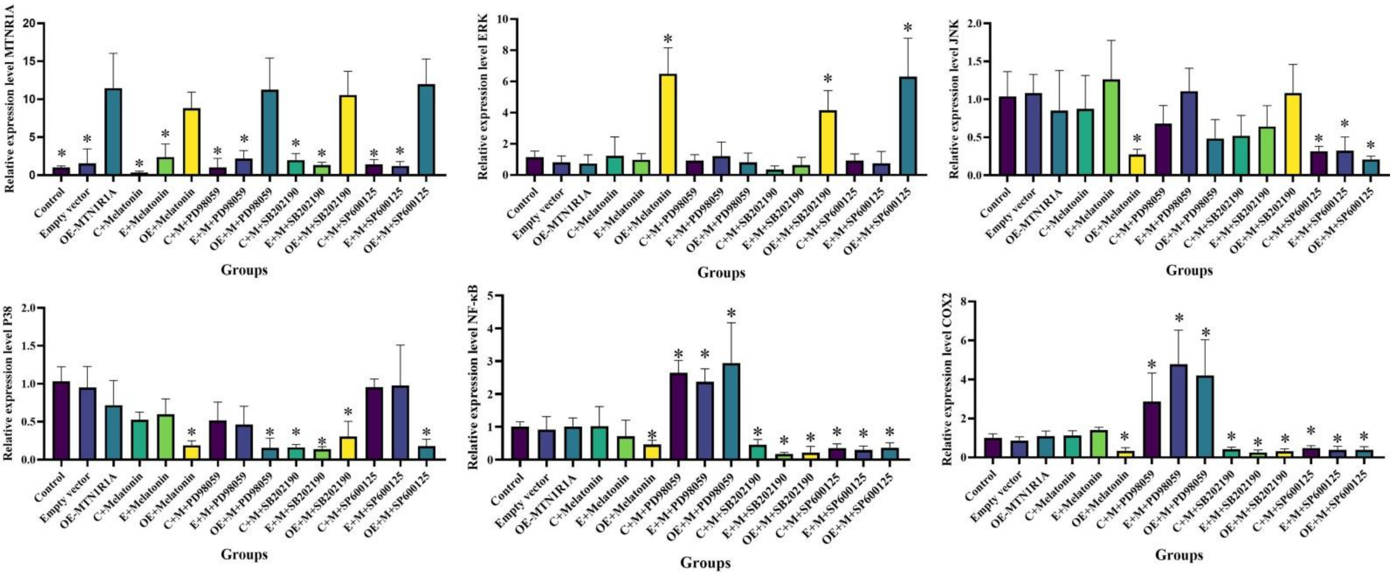

Fig. S3. Representative Western blot analysis showing the expression of proteins in different experimental groups. (A) β-actin (~42 kDa) for primary cells of *P. glenii*, (B) COX2 (~72 kDa), (C) NF-κB (~65 kDa), (D) β-actin (~65 kDa) for liver tissue of *P. glenii* during recovery from freezing, and (E) MTNR1A (~39 kDa). Protein bands were detected by chemiluminescence and quantified relative to β-actin.

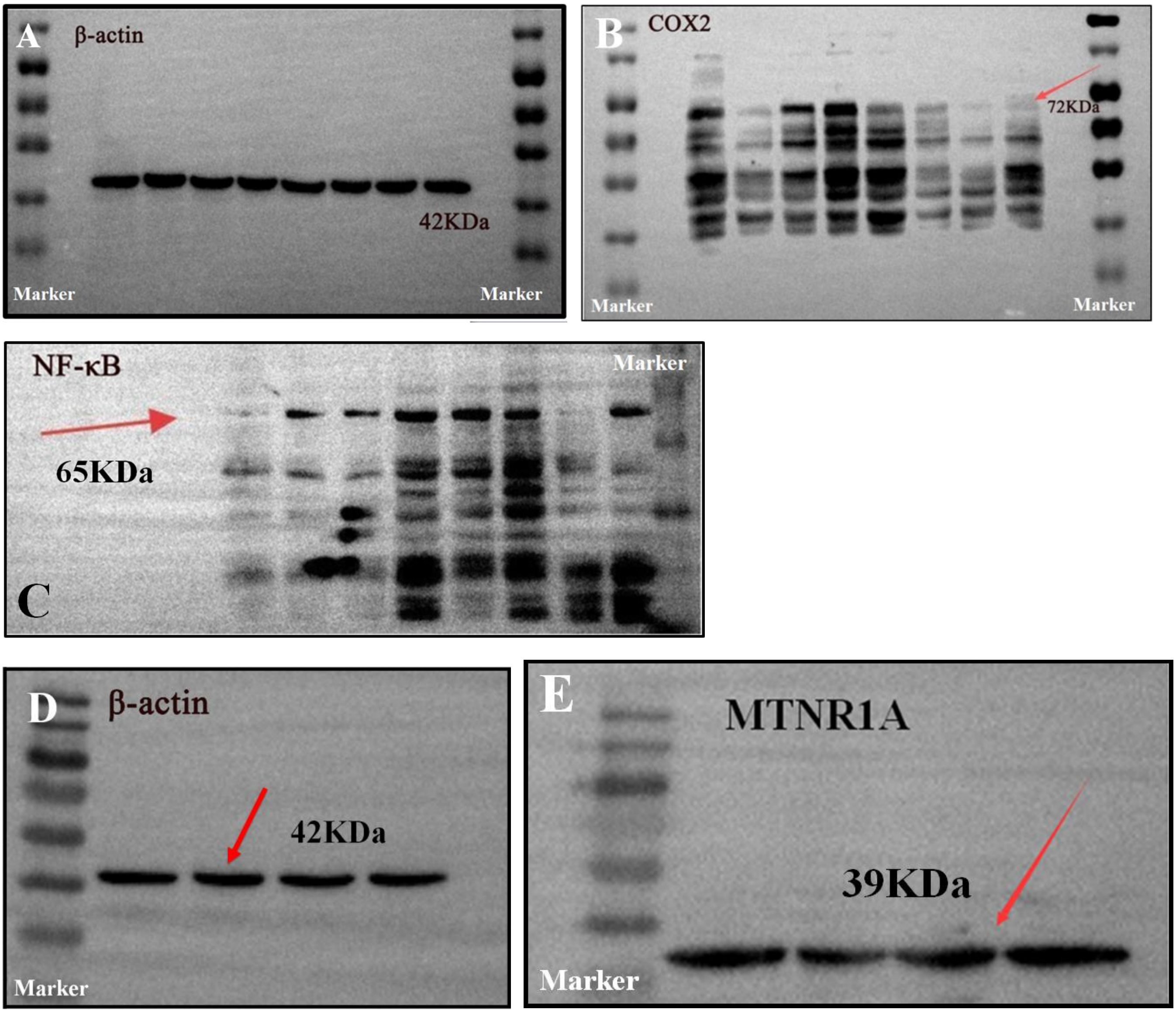

Table S1. Primers used in cloning and mRNA expression analyses.

Primers	Sequences (5′ –3′)	Accession number	Purpose used
MTNR1A-F	TTTTCCACTTCCTCGTC	NM_005958.4	Real-time quantitative PCR
MTNR1A-R	TTCAGTTTGGGTTTGCG		
ERK-F	GAGTCAAGCCAGAACAAC	D31661.1	
ERK-R	GTCAAAGTCAGCCTCATAGT		
JNK-F	TCTATCAGATGCTGTGTGGA	NM_001323302.2	
JNK-R	CACTACATAAGGCGTCATCA		
p38-F	GATATTTGGTCAGTGGGATG	XM_054354332.1	
p38-R	CTGTTAATGAGATAAGCGGG		
NF-κB-F	CACCCTGACCTTGCCTAT	NM_003998.4	
NF-κB-R	GCTGTCCTGTCCATTCTT		
COX2-F	GTTGCTGGTGGTAGGAA	AY462100.1	
COX2-R	TCAAGGAGAATGGTGCT		
β-actin -F	CAGGTCATCACCATTGG	DQ407611.1	
β-actin-R	TGGCGTACAGGTCTTTG		

Table S2. The accession numbers of all reference sequences used in the phylogenetic analysis.

MTNR1B	
Latin name	Accession number
Plectropomus leopardus	XP 042342800.1
Sphaeramia orbicularis	XP 029986598.1
Seriola dumerilii	XP 022181021.1
Centropristis striata	XP 051918797.1
Notolabrus celidotus	XP 034557415.1
Eleginops maclovinus	XP 063782291.1
Labrus mixtus	XP 060901938.1
Labrus bergylta	XP 029137029.1
Nerophis lumbriciformis	XP 061796982.1
Corythoichthys intestinalis	XP 057695683.1
Hippocampus zosterae	XP 051935015.1
Coregonus clupeaformis	XP 045076872.1
Salvelinus fontinalis	XP 055751203.1
Salvelinus namaycush	XP 038830051.1
Salmo salar	XP 045559914.1
Perccottus glenii	**XQU44399.1**
Boleophthalmus pectinirostris	XP 020783446.1
Periophthalmus magnuspinnatus	XP 033832853.2
Amphiprion ocellaris	XP 023123550.1
Scleropages formosus	XP 018604071.1
Megalops cyprinoides	XP 036392557.1
Lepisosteus oculatus	XP 006627897.1
MTNR1C	
Latin name	Accession number
Siniperca chuatsi	XP 044061901.1
Enoplosus armatus	XP 07070093.1
Brachyistius frenatus	XP 069552817.1
Embiotoca jacksoni	XP 069027063.1
Perca flavescens	XP 028444873.1
Sander lucioperca	XP 035859593.1
Paralichthys olivaceus	XP 019940363.1
Parametris bleekeri	XP 028271706.1
Lates calcarifer	XP 018534112.1
Thunnus thymus	XP 067455026.1
Echeneis naucrates	XP 029368136.1
Seriola dumerilii	XP 022607569.1
Chaetodon trifascialis	XP 070818607.1
Dicentrarchus labrax	XP 051276880.1
Laminichthys croceus	XP 010750373.1
Perccottus glenii	**XQU44583.2**
Sparus aurata	XP 030251478.1
MTNR1A	
Latin name	Accession number

Cyprinus carpio	XP 042594478.1
Danio rerio	NP 571468.1
Boleophthalmus pectinirostris	XP 020787058.1
Periophthalmus magnuspinnatus	XP 033830537.1
Perccottus glenii	**XQU44582.1**
Austrofundulus limnaeus	XP 013875638.1
Centropristis striata	XP 051919019.1
Brachyistius frenatus	XP 069545285.1
Eleginops maclovinus	XP 063739006.1
Lates calcarifer	XP 018558761.1
Sphaeramia orbicularis	XP 029986569.1
Pempheris kuenzingeri	XP 070712615.1
Microspathodon chrysurus	XP 035856961.1
Thunnus thymus	XP 067441157.1
Anabas testudineus	XP 026216726.1
Plectropomus leopardus	XP 042341131.1
Seriola dumerilii	XP 022593860.1
Scatophagus argus	XP 046274388.1
Epinephelus fuscoguttatus	XP 049427162.1
Notolabrus celidotus	XP 034544127.1
Semicossyphus pulcher	XP 009521486.1
Sparus aurata	XP 030251828.1
Callorhinchus milii	XP 042192782.1
